# Precision Medicine in Pediatric Nephrology: From Shared Clinical Phenotypes to Genotype-Guided Diagnosis and Management

**DOI:** 10.3390/genes17091069

**Published:** 2026-09-04

**Authors:** John Dotis, Nikoleta Printza

**Affiliations:** 1 Third Department of Pediatrics, Hippokration Hospital, Aristotle University of Thessaloniki, 54642 Thessaloniki, Greece; yandot@auth.gr; 2 First Department of Pediatrics, Hippokration Hospital, Aristotle University of Thessaloniki, 54642 Thessaloniki, Greece

**Keywords:** precision medicine, pediatric nephrology, monogenic kidney disease, next-generation sequencing, microscopic hematuria, cystic kidney disease, steroid-resistant nephrotic syndrome, thrombotic microangiopathy

## Abstract

Pediatric nephrology is shifting from broad phenotype-based labels toward molecularly defined, genotype-guided diagnosis and management. Childhood kidney disorders are enriched for monogenic causes, yet persistent microscopic hematuria, bilateral kidney cysts, steroid-resistant nephrotic syndrome, and thrombotic microangiopathy may represent shared endpoints of biologically distinct mechanisms. Using these four scenarios, this narrative review illustrates how structured phenotyping, pedigree analysis, biochemical evaluation, and appropriately selected genomic testing can establish etiology, revise diagnoses, and guide clinical decision-making. Molecular diagnosis may support early nephroprotection, prevent ineffective immunosuppression, reveal tumor or extrarenal risks, enable mechanism-based therapy, and optimize transplantation planning. It also enables cascade testing, reproductive counseling, presymptomatic evaluation of relatives, and safer assessment of living-related donors. Genomic findings, however, require clinical context. Variants must be evaluated against gene–disease validity, inheritance, segregation, molecular mechanism, and phenotype, while variants of uncertain significance should not independently determine treatment or donor eligibility. Negative or inconclusive findings should prompt phenotypic reassessment, evaluation of analytical limitations, targeted studies, and periodic genomic reanalysis. Emerging genome and long-read sequencing, multiomics, artificial intelligence, and RNA-based therapeutics may further expand precision care, but rigorous interpretation, equitable access, appropriate counseling, and multidisciplinary collaboration remain essential. Precision nephrology derives value not from identifying variants alone, but from converting molecular etiology into safer, anticipatory, and individualized care.

## 1. Introduction

Precision medicine seeks to move clinical care beyond broad diagnostic labels by integrating genomic, biochemical, environmental, and longitudinal data to identify the mechanism operating in an individual patient. This transition is particularly relevant to pediatric nephrology. Kidney disorders presenting in childhood are enriched for congenital and monogenic disease, begin before a complete syndromic phenotype has emerged, and evolve over decades. For affected children, the earliest findings such as microscopic hematuria, kidney cysts, proteinuria, or acute kidney injury (AKI), are common endpoints of biologically distinct disorders rather than disease-specific diagnoses [1,2].

Reliance on phenotype alone is therefore inadequate. The same urinary abnormality or imaging pattern may result from variants in genes affecting the glomerular basement membrane, podocyte, primary cilium, complement system, transcriptional regulation, or intracellular metabolism. Conversely, pathogenic variants within a single gene may produce variable age at onset, severity, extrarenal manifestations, and progression because of allelic heterogeneity, incomplete penetrance, modifier genes, and environmental triggers. Family history may be absent because of de novo variation, recessive inheritance, small pedigrees, or unrecognized disease in relatives [1,3]. Histology also has limitations: focal segmental glomerulosclerosis (FSGS) and thrombotic microangiopathy (TMA) describe patterns of injury rather than specific etiologic diagnoses and may therefore conceal distinct molecular mechanisms [4,5].

The availability of next-generation sequencing has transformed this diagnostic landscape. Gene panels, whole-exome sequencing (WES), and increasingly whole-genome sequencing (WGS) can replace sequential single-gene testing and shorten diagnostic trajectories. Across pediatric chronic kidney disease (CKD) cohorts, massively parallel sequencing yields a molecular diagnosis in approximately 30% of cases overall, with substantially higher yields in selected familial, syndromic, early-onset, cystic, tubular, or steroid-resistant (SRNS) phenotypes [1]. Large pediatric series demonstrate that diagnostic yield varies substantially according to clinical phenotype, age at presentation, cohort selection, referral strategy, and testing approach [3]. Sequencing may confirm a suspected diagnosis, refine its inheritance pattern, or completely reclassify a phenotype that had been assigned an acquired or nonspecific label [4,5].

The clinical value of molecular diagnosis extends beyond naming the disease. Genotype can determine the intensity of kidney and extrarenal surveillance, identify children unlikely to benefit from immunosuppression, support early nephroprotective treatment, reveal a metabolically treatable disorder, guide complement or enzyme replacement therapy, estimate recurrence risk after kidney transplantation, and improve living-donor assessment. It enables cascade testing, reproductive counseling, and presymptomatic evaluation of relatives [1,2]. In selected disorders, precision therapeutics target disease-relevant molecular pathways; for instance, genotype-guided RNA interference can suppress upstream transcripts within a specific metabolic pathway, shifting clinical management from non-specific supportive care toward mechanism-based disease modification [6].

Nevertheless, genomic data do not replace clinical reasoning. A pathogenic variant must be interpreted in the context of inheritance, phenotype, segregation, laboratory findings, and disease mechanism, while a variant of uncertain significance should not independently determine treatment. Negative testing does not exclude genetic disease because assays may miss structural, intronic, mosaic, epigenetic, or yet-unknown causes. Equitable implementation also requires appropriate consent, multidisciplinary expertise, periodic reinterpretation, and careful management of secondary findings [1,2,3]. Precision medicine is therefore best understood as an integrated diagnostic and therapeutic framework rather than sequencing alone.

Against this background, this narrative review examines how shared pediatric kidney phenotypes can lead to distinct molecular diagnoses, prognoses, and management pathways. Four illustrative scenarios—persistent microscopic hematuria, bilateral kidney cysts, SRNS, and TMA—are used to connect bedside findings with genotype-guided decision-making. By emphasizing clinically actionable contrasts, the review aims to show when genomic testing meaningfully changes surveillance, treatment, family evaluation, and long-term counseling, while also addressing current interpretive challenges and future directions in pediatric precision nephrology.

## 2. Review Approach

This narrative review examines how overlapping clinical phenotypes in pediatric nephrology translate into distinct molecular diagnoses and genotype-informed management strategies. The four clinical scenarios were predefined as representative phenotypes characterized by substantial diagnostic heterogeneity and clinically meaningful consequences of molecular diagnosis. They were selected, based on a combination of pediatric clinical relevance, diagnostic ambiguity, established or emerging genotype–phenotype associations, and the potential for molecular diagnosis to modify prognosis, surveillance, treatment, transplantation planning, or family counseling. Their selection was therefore not based on prevalence or diagnostic yield alone, but on their complementary value in illustrating distinct mechanisms and degrees of clinical actionability across pediatric nephrology.

Targeted literature searches were conducted in PubMed/MEDLINE and Google Scholar from database inception through 28 July 2026. The PubMed/MEDLINE search combined MeSH terms and free-text keywords related to “precision medicine,” “pediatric nephrology,” “genetic kidney disease,” “monogenic kidney disease,” “next-generation sequencing” (NGS, WES, and WGS), and the four target presentations: microscopic hematuria, cystic kidney disease, steroid-resistant nephrotic syndrome, and thrombotic microangiopathy. Phenotype-specific disease and gene terms, including “Alport syndrome,” “COL4A3,” “COL4A4,” “COL4A5,” “polycystic kidney disease,” “HNF1B,” “FSGS,” “genetic nephrotic syndrome,” “hemolytic uremic syndrome,” “atypical hemolytic uremic syndrome,” and “complement-mediated TMA,” were additionally used where relevant. Corresponding keyword combinations were used in Google Scholar. Reference lists of relevant clinical guidelines, consensus statements, major cohort studies, and key reviews were hand-searched to identify additional publications. No formal language restriction was applied; however, the evidence ultimately included in the narrative synthesis was predominantly published in English.

Priority was given to pediatric consensus guidelines, registry cohorts, clinical trials, primary genetic studies, and studies demonstrating genotype-specific management implications. Publications were considered particularly relevant when they provided information on molecular diagnosis, genotype–phenotype relationships, diagnostic yield, prognosis, surveillance, treatment response, transplantation, or clinically actionable management consequences. Adult data were included only when pediatric evidence was sparse but directly translatable. Mixed pediatric–adult populations were retained when pediatric-specific evidence could not be separated but the findings were considered applicable to pediatric practice. Case reports were included only when they described rare phenocopies or actionable molecular findings. Reports without a clinically relevant pediatric nephrology component or without sufficient information to support the diagnostic, prognostic, or therapeutic point under consideration were not prioritized for inclusion.

Titles and abstracts were initially assessed for relevance to the four predefined clinical scenarios, followed by full-text evaluation of potentially relevant publications. Duplicate records retrieved through more than one search route were considered only once. When multiple publications described overlapping cohorts, the most informative or updated report was preferentially used, while complementary reports were retained when they provided distinct clinical or longitudinal information. Evidence was qualitatively synthesized across the four predefined clinical scenarios, with pediatric evidence prioritized and adult-derived evidence identified where relevant, and all cited sources were manually cross-verified against the original publications and bibliographic records. No AI-generated, unverifiable, or non-existent references were retained, and generative-AI outputs were not used as independent sources of evidence. Because this was a narrative rather than a systematic review, retrieved and excluded records were not prospectively tracked in a PRISMA-style screening database; consequently, exact record-identification and exclusion counts are not reported.

## 3. From Clinical Phenotype to Molecular Diagnosis

### 3.1. Structured Phenotyping as the Starting Point

Precision nephrology integrates clinical reasoning with sequencing. The phenotype establishes pretest probability, while molecular findings may refine diagnosis, prognosis, and management. The process is iterative rather than linear. In a child with suspected inherited kidney disease, phenotyping should document presentation and age at onset, prenatal findings, disease course, blood pressure, kidney function, urinary findings, serum biochemical abnormalities, imaging, and histopathology. A pedigree spanning three generations should record unexplained kidney disease or kidney failure, consanguinity, and extrarenal findings. Assessment of hearing, vision, hematologic, neurodevelopmental, hepatopancreatic, and skeletal or dysmorphic features may reveal a syndromic diagnosis not apparent from the kidney phenotype alone [1,2,3].

Encoding granular observations with Human Phenotype Ontology terms supports computational phenotype matching and gene prioritization, particularly in multisystem disease [7]. Nevertheless, neither a negative family history nor the absence of a characteristic extrarenal feature should exclude a genetic etiology. Manifestations may be age-dependent or variably expressed. Molecular findings may prompt targeted evaluation or review of archived biopsy material to identify concordant features.

### 3.2. Selecting the Appropriate Molecular Test

The optimal testing strategy depends on the clinical question and technical characteristics of the suspected locus. Age at onset, family history, consanguinity, syndromic or extrarenal features, and the breadth of the differential diagnosis should also inform test selection. Targeted testing is appropriate when a familial pathogenic variant is known. For a recognizable but genetically heterogeneous presentation, a curated kidney gene panel may provide deep coverage and copy number analysis at exon level while limiting unrelated findings. Exome sequencing may be preferred when the differential is broad, the presentation is atypical or multisystemic, or a panel is unrevealing; it also permits reanalysis as knowledge of disease genes expands. Trio-based exome sequencing may be particularly useful in early-onset, syndromic, apparently de novo, or consanguineous presentations. Genome sequencing provides more uniform coverage and may improve detection of structural, intronic, and other variants, although performance depends on the analytical pipeline and interpretive evidence [1,2,4].

No sequencing platform reliably captures every relevant variant class or disease mechanism. Copy number analysis is essential when deletions or duplications are plausible, including recurrent 17q12 deletions encompassing Hepatocyte Nuclear Factor 1-Beta (HNF1B). Polycystic Kidney Disease 1 (PKD1) regions homologous to pseudogenes and complex Complement Factor H/Complement Factor H-Related (CFH/CFHR) rearrangements may require validated methods tailored to specific loci. Mitochondrial DNA variants, low-level mosaicism, and deep intronic variants may likewise require dedicated assays. Accordingly, suspected copy-number variation or technically challenging loci should prompt use of appropriately validated methods rather than relying on standard sequencing alone. When a treatable metabolic disorder is suspected, biochemical or enzymatic studies should proceed in parallel with sequencing. Trio analysis, when the child and both biological parents are available, strengthens evaluation of de novo occurrence, recessive inheritance, phasing, and segregation [1,2]. After nondiagnostic first-tier testing, the phenotype and analytical limitations should be reassessed before proceeding to broader sequencing or dedicated complementary assays.

### 3.3. From Variant Detection to a Molecular Diagnosis

Pretest counseling should address diagnostic, negative, and uncertain results; secondary findings, where applicable; and implications for relatives. A rare variant does not establish disease causation. Interpretation should follow standardized American College of Medical Genetics and Genomics/Association for Molecular Pathology (ACMG/AMP) criteria integrating population, computational, functional, segregation, and allelic evidence with the disease mechanism, producing the categories pathogenic, likely pathogenic, uncertain significance, likely benign or benign [8]. Even a pathogenic or likely pathogenic classification is insufficient alone. The finding must be compatible with the validated association between the gene and disease, molecular mechanism, inheritance pattern, zygosity, penetrance, and phenotype. Incomplete penetrance, variable expressivity, and hypomorphic alleles may substantially modify the relationship between genotype and clinical phenotype, while cis/trans phasing can be critical when evaluating recessive disease or multiple variants within the same gene. Phenocopies should also be considered when the molecular finding does not fully explain the clinical presentation. Population-frequency evidence requires particular caution in ancestry groups that remain underrepresented in genomic reference databases. A variant of uncertain significance should not independently guide treatment, predictive testing of unaffected relatives, or living donor eligibility; testing relatives may support segregation analysis. Functional studies and phenotypic reassessment may support reclassification. Because databases and disease associations evolve, reinterpretation should be considered when the phenotype progresses or new family information becomes available. Variant classification may therefore change over time as additional population, segregation, functional, or disease-specific evidence becomes available.

### 3.4. Converting Molecular Diagnosis into Clinical Action

The endpoint of testing is an actionable clinical and molecular synthesis, not merely a laboratory label. In a prospective cohort of 204 children and adults with suspected monogenic kidney disease, WES established a diagnosis in 39%; among diagnosed patients, the clinical diagnosis changed in 39% and management was influenced in 59% [9]. Clinical action may reclassify an imprecise diagnosis, initiate targeted or nephroprotective treatment, avoid ineffective immunosuppression, define kidney and extrarenal surveillance, estimate recurrence risk after transplantation, and inform assessment of prospective living related donors [5]. Definitive results may also inform cascade testing, reproductive counseling, and presymptomatic evaluation of relatives [1,2,9].

**Figure 1:**
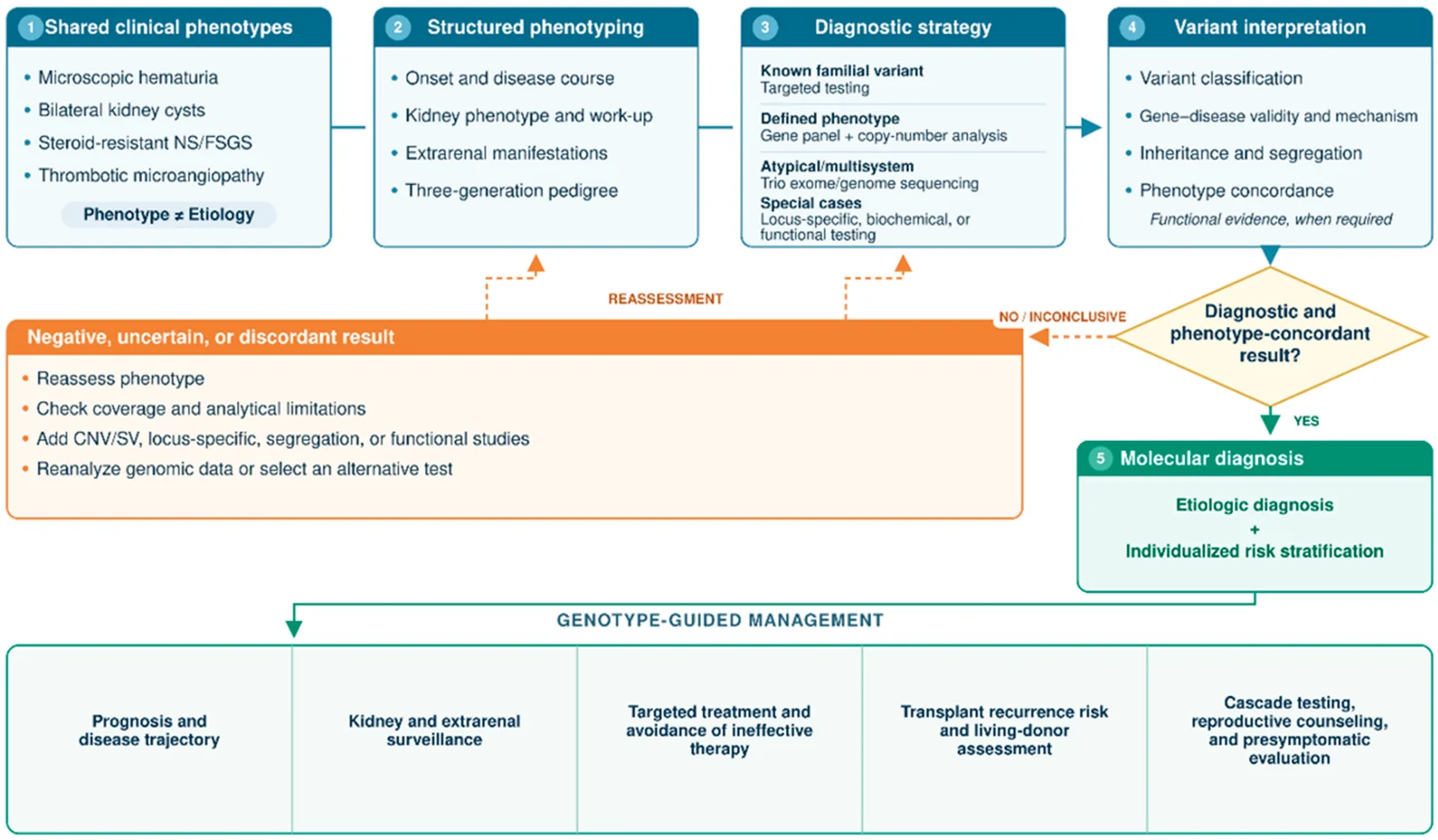
From shared clinical phenotypes to genotype-guided management. Structured phenotyping informs test selection and variant interpretation, enabling phenotype-concordant molecular diagnoses and individualized management. Negative, uncertain, or discordant results prompt reassessment and genomic reanalysis. CNV, copy-number variant; FSGS, focal segmental glomerulosclerosis; NS, nephrotic syndrome; SV, structural variant.

A negative or uncertain result, or one discordant with the phenotype, should not close the diagnostic process. It should prompt reassessment of phenotype, analytical limitations, copy number and structural variant detection, inheritance, and phenocopies. When relevant, phasing, segregation, ancestry-specific population data, and the possibility of reduced penetrance or variable expressivity should also be reconsidered. Segregation or functional studies, assays tailored to specific loci, genomic reanalysis, or alternative testing may be appropriate. This iterative pathway from a shared phenotype through appropriate testing and interpretation to molecular diagnosis and management guided by genotype is summarized in Figure 1 and frames the four clinical scenarios that follow. In the phenotype-specific sections that follow, diagnostic associations, prognostic evidence, established or guideline-supported management, and emerging or investigational approaches are distinguished according to the maturity of the available evidence.

## 4. The Child with Persistent Microscopic Hematuria

### 4.1. Illustrative Clinical Vignette

An 8-year-old boy is referred after microscopic hematuria is documented in three separate first-morning urine samples over six months. Urine microscopy shows dysmorphic erythrocytes, whereas blood pressure, estimated glomerular filtration rate (eGFR), urinary albumin-to-creatinine ratio (UACR), and kidney ultrasonography are normal. He has no systemic symptoms or visible hematuria. A maternal aunt reportedly has longstanding microscopic hematuria, although no relative has received a defined kidney diagnosis. This presentation illustrates the central problem: persistent glomerular hematuria may be the earliest and, for years, the only manifestation of disorders with markedly different inheritance, prognosis, extrarenal complications, and management.

### 4.2. Phenotypic Overlap and Key Genetic Diagnoses

Persistent microscopic hematuria should be confirmed by repeated urinalyses over 3–6 months [10]. After excluding transient causes, hypercalciuria, urinary tract disease, and acquired glomerulonephritis, a monogenic disorder should be considered even when kidney function and albumin excretion are normal [10,11]. In contemporary pediatric cohorts, pathogenic or likely pathogenic variants in the genes encoding the α3, α4, and α5 chains of type IV collagen (*COL4A3*, *COL4A4*, and *COL4A5*, respectively) have been identified in a substantial proportion of children with persistent hematuria. In one single-center series, such variants were identified in 91 of 134 tested children and young people (68%); importantly, only approximately half of genetically diagnosed patients had a relevant family history [12]. Other cohorts have reported lower but still clinically meaningful yields, reflecting differences in referral criteria and testing strategies [13,14].

The principal diagnostic group is the type IV collagen disease spectrum. Hemizygous *COL4A5* variants cause X-linked Alport syndrome (XLAS), whereas biallelic *COL4A3* or *COL4A4* variants cause autosomal recessive Alport syndrome (ARAS). Heterozygous *COL4A3* or *COL4A4* variants frequently present as isolated hematuria, historically labelled “thin basement membrane nephropathy” or “benign familial hematuria.” The latter term should be abandoned because albuminuria, hypertension, and CKD may emerge later, although penetrance and absolute risk are substantially lower than estimates derived from highly selected families [11,15]. In a mixed pediatric–adult registry cohort of 294 patients with heterozygous disease-causing *COL4A3/4* variants, each five-year shift toward younger disease onset was independently associated with a 64% higher risk of albuminuria and a 34% higher risk of CKD grade 2, identifying age at onset as a potential risk-stratification marker [16]. In a clinically ascertained cohort, 17% developed proteinuria and 7.5% progressed to CKD or kidney replacement therapy, although referral-based ascertainment limits extrapolation to unselected variant carriers [17]. These data argue against a uniformly benign label without implying uniformly high penetrance. They also support a unified, genotype-driven nomenclature in which collagen IV nephropathies are classified by the affected gene, inheritance pattern, and evolving clinical risk rather than by an early phenotypic snapshot [18].

Two less common diagnoses are especially informative. CFHR5 nephropathy is an autosomal dominant complement 3 (C3) glomerulopathy caused classically by an internal duplication of *CFHR5* exons 2–3 in individuals of Cypriot ancestry [19,20]. It often begins with persistent microscopic hematuria and infection-associated macroscopic hematuria; proteinuria and progressive kidney impairment may appear later, with a strikingly higher risk in males. In myosin heavy chain 9 (*MYH9*)-related disease, hematuria can coexist with congenital macrothrombocytopenia and later proteinuria, sensorineural hearing loss, cataracts, or elevated liver enzymes [21,22]. Automated analyzers may underestimate platelet count and size because giant platelets are not recognized correctly [22]. Identifying this clue prevents the hematuria from being misclassified as an isolated collagen IV disorder and avoids inappropriate immunosuppression or splenectomy.

### 4.3. Clinical Clues and Genetic Testing Strategy

Evaluation should first establish glomerular origin and quantify risk. Repeated urinalysis with microscopy, first-morning UACR or protein-to-creatinine ratio, blood pressure, serum creatinine/eGFR, and a three-generation pedigree are essential. The pedigree should capture hematuria, kidney failure of unknown cause, hearing loss, ocular disease, and male-versus-female severity. A complete blood count must be interpreted with platelet size and a peripheral smear when thrombocytopenia, easy bruising, or analyzer flags raise suspicion for *MYH9*-related disease. Complement studies are appropriate when C3 glomerulopathy is suspected, although normal circulating complement does not exclude tissue-restricted alternative-pathway dysregulation. Recurrent visible hematuria after respiratory infections and Cypriot ancestry should specifically prompt consideration of *CFHR5*.

For persistent glomerular hematuria, simultaneous analysis of *COL4A3*, *COL4A4*, and *COL4A5* is preferred over sequential single-gene testing [10,11,15]. Testing should detect single-nucleotide and splice variants as well as exon-level deletions, duplications, and other structural variants. A broader kidney disease panel that includes *CFHR5* and *MYH9* is efficient when the phenotype is not syndromically distinctive; exome or genome sequencing is appropriate after a negative panel or when the presentation is atypical. A pathogenic or likely pathogenic result can provide a molecular diagnosis without kidney biopsy in many children. Biopsy remains valuable when testing is negative or inconclusive, the course is unexpectedly inflammatory or rapid, or a superimposed glomerular disease is suspected. A variant of uncertain significance should not direct care in isolation; segregation analysis, phenotype reassessment, and periodic reinterpretation are required.

### 4.4. Genotype-Specific Prognostic and Management Implications

The molecular result changes both the intensity and content of follow-up. Risk is not uniform across collagen IV nephropathies: males with COL4A5-related XLAS and patients with biallelic COL4A3/COL4A4-related ARAS generally carry the highest risk of progressive kidney disease and extrarenal complications, whereas heterozygous COL4A3/COL4A4-related disease shows substantially more variable penetrance and, on average, a lower risk requiring proportionate surveillance rather than equivalently intensive management. Boys with XLAS and children of either sex with ARAS require particularly close longitudinal follow-up for progressive albuminuria, kidney function loss, hearing impairment, and characteristic ocular abnormalities. Current European guidance recommends initiating and titrating renin–angiotensin system (RAS) blockade when microalbuminuria develops, irrespective of genetic subgroup, and considering treatment from the stage of isolated microscopic hematuria after age two years in males with XLAS and in children with ARAS, providing guideline-supported rationale for early nephroprotection in these higher-risk groups [11]. This represents guideline-supported management. The pediatric EARLY PRO-TECT Alport trial demonstrated that ramipril was safe and provided supportive evidence that early therapy delays disease progression, although limited recruitment reduced statistical precision [23]. Complementary registry evidence in 101 patients with ARAS found that earlier RAS inhibition was associated with later CKD grade 5; no patient treated from the microalbuminuria stage progressed to CKD grade 5 during observation, although these observational data should not be interpreted as equivalent to randomized evidence [24]. These children also require genotype- and age-adapted audiologic and ophthalmologic surveillance.

By contrast, a girl with XLAS or a child with a heterozygous *COL4A3* or *COL4A4* variant usually has a lower short-term risk, but the diagnosis is not “benign.” Follow-up should include blood pressure, UACR, and eGFR; RAS blockade is indicated when albuminuria or hypertension emerges, rather than solely for isolated hematuria in heterozygous autosomal disease [11]. Unexpected nephrotic-range proteinuria or rapid eGFR decline should trigger reassessment for an additional kidney disorder instead of being automatically attributed to the collagen variant.

In *CFHR5* nephropathy, the diagnosis redirects counseling toward autosomal dominant transmission, sex-modified prognosis, serial monitoring of proteinuria, blood pressure, eGFR, and evaluation of relatives. Management remains principally supportive, including RAS blockade for albuminuria or hypertension; complement inhibition is biologically attractive but cannot yet be considered established genotype-specific therapy [25]. In *MYH9*-related disease, care becomes multidisciplinary. Kidney surveillance focuses particularly on proteinuria, while hematology input informs bleeding risk and the safety of biopsy or surgery; audiologic and ophthalmologic follow-up and avoidance of platelet-inhibiting drugs are also relevant [21,22]. Molecular confirmation enables targeted testing of relatives and prevents ineffective immune therapy.

Returning to the illustrative vignette, identification of a hemizygous *COL4A5* variant would support early RAS blockade, audiologic surveillance, and cascade testing of relatives, whereas a heterozygous *COL4A3* or *COL4A4* variant in the absence of albuminuria or hypertension would favor structured longitudinal monitoring, with treatment introduced if additional risk markers emerge. The principal genotype-specific distinctions and management pathways are summarized in Table 1.

### 4.5. Precision Medicine Take-Home Message

Persistent microscopic hematuria is a phenotype rather than a diagnosis. Molecular testing can distinguish collagen IV disease and other inherited causes with different prognostic, surveillance, and family-counseling implications, allowing follow-up and treatment intensity to be tailored to the underlying diagnosis.

## 5. The Child with Bilateral Kidney Cysts

### 5.1. Illustrative Clinical Vignette

A 5-year-old girl is referred for bilateral echogenic, cystic kidneys first detected during prenatal ultrasonography. Postnatal ultrasonography confirms bilateral cystic abnormalities but remains etiologically nonspecific. Blood pressure, eGFR, and UACR are normal. No hepatic, ophthalmologic, metabolic, skeletal, or neurodevelopmental abnormalities are recognized. Her parents have no known kidney disease, although neither has undergone kidney imaging. This presentation illustrates the central problem: bilateral kidney cysts are an imaging phenotype that may reflect disorders with different inheritance, extrarenal involvement, prognosis, and management.

### 5.2. Phenotypic Overlap and Key Genetic Diagnoses

In children, “cystic kidneys” encompass true macrocysts, collecting-duct ectasia, microcystic hyperechogenicity, glomerulocystic change, and cystic renal dysplasia. Kidney size, corticomedullary differentiation, cyst distribution, urinary tract abnormalities, and liver findings narrow the differential but rarely provide molecular certainty [26,27]. An apparently negative family history has limited exclusionary value: *HNF1B* and paired box 2 (*PAX2*) variants may arise de novo, polycystic kidney and hepatic disease 1 (*PKHD1*)-related disease is recessive, and a parent with autosomal dominant polycystic kidney disease (ADPKD) may remain unrecognized. Pediatric data confirm that integrating imaging with genomic findings frequently revises the initial clinical classification [28].

*HNF1B*-related disease is a leading monogenic cause of developmental kidney abnormalities. Its renal spectrum includes prenatal hyperechogenicity, bilateral or unilateral cysts, hypoplasia or dysplasia, a solitary kidney, and other congenital anomalies of the kidney and urinary tract (CAKUT). Hypomagnesemia due to renal magnesium wasting, hyperuricemia, early-onset diabetes, pancreatic hypoplasia or exocrine dysfunction, abnormal liver enzymes, and genital tract anomalies provide diagnostic clues [29,30]. The recurrent 17q12 deletion includes *HNF1B* but may confer neurodevelopmental, behavioral, or psychiatric risk. A large mixed pediatric–adult cohort found slower progression to CKD grade 3 with the 17q12 deletion than with intragenic *HNF1B* variants, whose location also influenced kidney survival [31].

*PKHD1*-related autosomal recessive polycystic kidney disease (ARPKD) classically produces enlarged hyperechogenic kidneys, reduced corticomedullary differentiation, and radially oriented collecting-duct dilatation; discrete macrocysts may be inconspicuous early. The phenotype ranges from perinatal respiratory compromise to childhood-onset hypertension and progressive CKD. Congenital hepatic fibrosis is integral, although portal hypertension, splenomegaly, varices, or recurrent cholangitis may emerge after the renal presentation [32,33,34].

By contrast, heterozygous *PKD1* or *PKD2* variants cause typical ADPKD. Cysts usually develop within otherwise preserved parenchyma, and hypertension may precede measurable loss of eGFR. *PKD1* generally confers a more severe course than *PKD2*, but substantial intrafamilial variability limits deterministic prediction. Very-early-onset or unexpectedly severe disease should prompt consideration of hypomorphic or biallelic *PKD1* alleles, variants in more than one cystic-disease gene, or a tuberous sclerosis complex 2 (*TSC2*)–*PKD1* contiguous-gene deletion [35,36,37]. Pediatric interventional evidence is also available from a randomized controlled trial evaluating tolvaptan in children and adolescents with ADPKD [38].

Intraflagellar transport 140 (*IFT140*) demonstrates why allelic state must be incorporated into diagnosis. Monoallelic loss-of-function variants cause an atypical ADPKD-spectrum phenotype, often with fewer large cysts, limited kidney enlargement, and a generally favorable kidney prognosis; however, prenatal and childhood-onset presentations are now documented [39,40,41]. Biallelic *IFT140* variants instead cause a recessive syndromic ciliopathy, including Mainzer–Saldino or Jeune-spectrum disease, with possible retinal dystrophy, skeletal abnormalities, and progressive kidney dysfunction [42]. Finally, *PAX2*-related disease may resemble a cystic disorder when cysts accompany renal hypodysplasia or broader CAKUT. Small dysplastic kidneys, proteinuria, reduced nephron mass, and optic nerve coloboma or dysplasia are particularly informative; hearing impairment may also occur [43,44,45]. Evidence defining the monoallelic IFT140 phenotype derives largely from mixed-age or adult-enriched cohorts, although childhood-onset disease has also been specifically documented [39,40,41].

### 5.3. Clinical Clues and Genetic Testing Strategy

Evaluation should first refine the phenotype. The prenatal record should be reviewed for gestational age at detection, kidney growth, oligohydramnios, and pulmonary compromise. Ultrasonography should document kidney size and symmetry, echogenicity, corticomedullary differentiation, cyst number, size and distribution, collecting-system abnormalities, liver echotexture, biliary abnormalities, and splenomegaly [26,27]. A three-generation pedigree should capture kidney cysts, early hypertension, kidney failure, diabetes, gout, intracranial aneurysm, visual or hearing impairment, and consanguinity. Baseline assessment includes standardized blood pressure, serum creatinine/eGFR, UACR or protein-to-creatinine ratio, magnesium and other electrolytes, uric acid, glucose or HbA1c, and liver enzymes. Ophthalmologic evaluation is indicated when *PAX2* disease is plausible; retinal, skeletal, and developmental assessment is appropriate when a biallelic ciliopathy is suspected.

When imaging is not pathognomonic, a broad cystic-kidney/CAKUT panel is more efficient than sequential single-gene testing [28]. The assay should detect single-nucleotide and splice variants, exon-level deletions or duplications, and larger copy-number changes; it must include technically validated analysis of the pseudogene-rich regions of *PKD1* (e.g., long-range PCR-based sequencing or custom long-read pipelines) and dedicated detection of *HNF1B* deletions and the recurrent 17q12 deletion. Chromosomal microarray is useful when 17q12 deletion and neurodevelopmental features coexist. Trio-based exome or genome sequencing is appropriate after a negative panel or for syndromic or atypical presentations. Segregation analysis and parental imaging can clarify inheritance, but a variant of uncertain significance should not determine prognosis or treatment alone. Kidney biopsy is reserved for unresolved cases and suspected superimposed disease; it is not the primary etiologic test for a stable bilateral cystic phenotype.

### 5.4. Genotype-Specific Prognostic and Management Implications

The molecular result changes both kidney follow-up and the search for extrarenal disease. In *HNF1B*-related disease, surveillance extends beyond blood pressure, UACR, and eGFR to serum magnesium, uric acid, glucose or HbA1c, liver enzymes, pancreatic disease, and genital tract anomalies. A 17q12 deletion additionally warrants developmentally appropriate neurocognitive and behavioral assessment. Recent genotype–phenotype data support more individualized kidney prognostication, although the molecular class cannot replace longitudinal clinical monitoring [29,31].

For *PKHD1*-related ARPKD, control of hypertension and management of CKD must be integrated with lifelong hepatology surveillance. Clinical review should address splenomegaly, thrombocytopenia as a marker of hypersplenism, portal hypertension, variceal risk, and recurrent cholangitis. Advanced disease may require coordinated decisions regarding isolated kidney or liver transplantation versus combined liver–kidney transplantation [33,34]. In *PKD1*/*PKD2*-related ADPKD, follow-up prioritizes standardized blood pressure assessment, ambulatory monitoring when indicated, urine albumin excretion, kidney function, healthy lifestyle measures, and clinically purposeful rather than indiscriminate serial imaging [36,37]. Blood-pressure control and RAS blockade with an angiotensin-converting enzyme inhibitor (ACEi) or angiotensin receptor blocker (ARB) as first-line therapy for hypertension represent established supportive management in pediatric ADPKD. A pediatric randomized trial demonstrated pharmacodynamic activity of tolvaptan with manageable aquaretic effects, but current evidence is insufficient for routine disease-modifying treatment with tolvaptan in children with ADPKD. Tolvaptan is currently authorized for ADPKD only in adults in both the European Union and the United States; its use for pediatric ADPKD therefore remains off-label with respect to this indication [37,38].

In monoallelic *IFT140*-related disease, counseling should reflect an atypical, usually milder ADPKD-spectrum trajectory rather than automatically importing the prognosis of classic *PKD1*-ADPKD. Conversely, biallelic *IFT140* disease requires multidisciplinary ciliopathy care, including ophthalmologic and skeletal surveillance [39,40,42]. For *PAX2*-related disease, follow-up resembles that for renal hypodysplasia, with attention to eGFR, proteinuria, and blood pressure; formal ophthalmologic examination is required even in the absence of visual symptoms, and audiology may be appropriate [44,45]. Across diagnoses, molecular confirmation enables targeted testing of relatives, accurate recurrence-risk counseling, and avoidance of surveillance that belongs to a phenotypically similar but biologically different disorder.

Returning to the illustrative vignette, identification of an *HNF1B* variant or 17q12 deletion would redirect care toward metabolic, pancreatic, hepatic, genital, and when appropriate, neurodevelopmental surveillance; biallelic *PKHD1* variants would establish an immediate kidney–liver pathway with portal-hypertension monitoring and autosomal-recessive counseling; whereas a monoallelic *IFT140* variant would support an atypical ADPKD-spectrum diagnosis without automatically implying a syndromic ciliopathy. Thus, the same prenatal and postnatal ultrasound phenotype would lead to substantially different monitoring, prognostic counseling, and family evaluation. The principal genotype-specific distinctions and management pathways are summarized in Table 2.

### 5.5. Precision Medicine Take-Home Message

Bilateral kidney cysts represent a shared imaging phenotype with genetically distinct causes. Molecular diagnosis can redefine prognosis, extrarenal surveillance, reproductive counseling, and, in selected disorders, management.

## 6. The Child with Steroid-Resistant Nephrotic Syndrome

### 6.1. Illustrative Clinical Vignette

A 4-year-old boy presents with generalized edema, nephrotic-range proteinuria, hypoalbuminemia, and hyperlipidemia. Blood pressure and eGFR are initially normal, complement levels are preserved, and no secondary cause is identified. He does not achieve complete remission after 4 weeks of oral glucocorticoids and remains proteinuric during the subsequent confirmation period. Kidney biopsy shows FSGS. There is no known family history of kidney disease, consanguinity, genital anomaly, ocular abnormality, hearing impairment, or neurologic involvement. The central question is whether this apparently uniform SRNS reflects an immune-mediated podocytopathy or one of several genetic disorders requiring fundamentally different management.

### 6.2. Phenotypic Overlap and Key Genetic Diagnoses

SRNS is defined by treatment response, not by a single pathobiological mechanism. Approximately 10–15% of children with idiopathic nephrotic syndrome fail to achieve complete remission with glucocorticoids, and a monogenic cause is identified in 10–30%, with the probability highest in congenital, infantile, familial, consanguineous, or syndromic disease [46,47]. Histologic findings such as FSGS or diffuse mesangial sclerosis (DMS) narrow the differential but do not establish etiology. In the PodoNet registry and a United Kingdom national cohort, pathogenic variants in *NPHS1*, *NPHS2* and *WT1* were among the most frequently identified genetic causes of SRNS, highlighting their importance in European and European-led pediatric cohorts [48,49].

Biallelic *NPHS1* variants disrupt nephrin, a central component of the slit diaphragm. Although classically associated with congenital nephrotic syndrome, hypomorphic variants can cause infantile or childhood-onset SRNS. Severe proteinuria, prematurity, placentomegaly, or elevated maternal serum alpha-fetoprotein may suggest early disease, but later presentations may lack distinctive clues [50]. Biallelic *NPHS2* variants impair podocin and commonly produce childhood or adolescent SRNS with FSGS. The phenotype is usually kidney-limited; an apparently negative family history is compatible with recessive inheritance, and age at onset varies with the allelic combination.

Heterozygous *WT1* variants may cause isolated SRNS or a broader disorder encompassing DMS or FSGS, rapid CKD progression, Wilms tumor predisposition, and differences in sex development. Classic Denys–Drash and Frasier labels capture only part of a continuous genotype-dependent spectrum, and external genitalia may appear normal. Consequently, *WT1* disease cannot be excluded by the absence of tumor or a recognized syndromic phenotype at presentation [51]. Biallelic *LAMB2* variants cause Pierson syndrome, typically combining congenital or infantile nephrotic syndrome with microcoria. Hypomorphic variants may present later with less conspicuous ocular disease, while retinal, neurologic, or neuromuscular abnormalities may evolve over time [52].

*INF2* occupies a different age and inheritance category. Evidence derives predominantly from familial mixed-age cohorts, in which heterozygous pathogenic variants predominantly cause autosomal dominant familial FSGS of adolescent or adult onset; childhood SRNS is possible but is not the typical presentation. Some variants also cause Charcot–Marie–Tooth neuropathy, making distal weakness, sensory loss, gait abnormalities, or a combined kidney–neurologic family history diagnostically informative [53].

Recessive defects in coenzyme Q10 (CoQ10) biosynthesis define a small but therapeutically critical subgroup. *COQ8B*, historically termed *ADCK4*, often causes insidious proteinuria and FSGS in older children or adolescents; *COQ2*, *COQ6*, *PDSS1*, *PDSS2*, and *COQ9* can produce earlier or multisystem disease. Hearing loss, seizures, developmental regression, ataxia, myopathy, or retinopathy may indicate mitochondrial involvement, although kidney-limited disease is common [54,55].

Type IV collagen–related nephropathies constitute an important genetic cause of proteinuric kidney disease that may present as apparent SRNS or FSGS. Variants in *COL4A3*, *COL4A4*, and *COL4A5* can produce a phenotype dominated by proteinuria and FSGS, even when classical Alport-associated features are absent at initial presentation. Establishing a pathogenic molecular diagnosis distinguishes secondary glomerular injury from a primary podocytopathy and redirects management away from ineffective immunosuppression toward nephroprotective treatment, genotype-appropriate surveillance, and family screening [56,57].

### 6.3. Clinical Clues and Genetic Testing Strategy

Evaluation should confirm primary SRNS, verify adherence and glucocorticoid exposure, and exclude infection, systemic disease, medication-related injury, and other secondary causes. Age at onset, a three-generation pedigree, consanguinity, prenatal history, kidney-function trajectory, and the presence or absence of post-transplant recurrence in affected relatives may help distinguish monogenic structural disease from presumed circulating-factor disease [47]. Examination should address ocular findings, hearing, neurologic function, growth, external genitalia, and urogenital abnormalities. Kidney biopsy remains valuable for defining the lesion, excluding immune-complex disease, and assessing chronicity, but FSGS must be interpreted as a pattern of injury rather than a molecular diagnosis. By contrast, DMS, particularly in congenital or infantile presentations, should heighten suspicion of a monogenic etiology [47,51,52].

Genetic testing should begin early in primary SRNS, alongside clinical and pathologic evaluation [58,59,60]. A comprehensive glomerulopathy panel should include podocyte, basement-membrane, mitochondrial, and syndromic genes and detect single-nucleotide and splice variants together with exon-level and larger copy-number changes. Trio-based exome or genome sequencing is appropriate after a nondiagnostic panel or for an atypical or multisystem phenotype. Broader recommendations for clinical genetic testing in CKD likewise emphasize precise phenotyping, validated variant interpretation, and genetic counseling [1,2]. Segregation analysis may clarify causality, but a variant of uncertain significance should not independently justify withdrawal of effective therapy, tumor surveillance, or major reproductive decisions. More broadly, treatment decisions after a molecular diagnosis should integrate genotype with prior treatment response, histopathologic findings, kidney function, chronicity, and disease stage rather than rely on the genetic result in isolation.

### 6.4. Genotype-Specific Prognostic and Management Implications

The molecular result separates children likely to benefit from intensified immunosuppression from those requiring supportive, surveillance-based, or metabolic treatment. In presumed immune-mediated SRNS, a calcineurin inhibitor remains recommended first-line therapy after confirmation of steroid resistance. Conversely, a confirmed monogenic structural podocytopathy generally argues against prolonged ineffective immunosuppression, particularly when no meaningful response is observed [47,61]. However, molecular diagnosis does not automatically mandate withdrawal of immunosuppression; treatment decisions should integrate genotype with documented treatment response, biopsy findings, kidney function, chronicity, and disease stage. This recommendation is supported primarily by guideline/consensus guidance and observational evidence rather than randomized genotype-stratified trials. Observational cohorts demonstrate substantially greater calcineurin-inhibitor responsiveness in nongenetic than genetic disease [62]. Across genotypes, management includes blood-pressure control, RAS blockade when tolerated, reduction in proteinuria, prevention of nephrotic complications, and longitudinal assessment of eGFR.

For *NPHS1* and *NPHS2* disease, diagnosis supports autosomal-recessive counseling and avoids serial empirical immunosuppressive regimens. The risk of true post-transplant recurrence is generally very low in monogenic SRNS. However, patients with biallelic NPHS1 variants causing complete nephrin deficiency may develop a distinct post-transplant alloimmune anti-nephrin glomerulopathy rather than recurrence of the original genetic disease [50]. A *WT1* diagnosis requires genotype- and age-adapted Wilms tumor surveillance and coordinated assessment of sex chromosomes, internal reproductive anatomy, gonadal development, and gonadoblastoma risk. These implications apply even when external genitalia are unremarkable [51].

*LAMB2* disease mandates formal ophthalmologic assessment, including evaluation for subtle microcoria and retinal abnormalities, together with neurologic and developmental surveillance. An *INF2* diagnosis shifts counseling toward dominant inheritance, testing of relatives, and neurologic assessment for Charcot–Marie–Tooth disease. In genetically confirmed primary CoQ10 deficiency, early and sustained CoQ10 supplementation should be initiated because observational evidence associates treatment with reduced proteinuria and better preservation of kidney function, whereas delayed treatment after advanced scarring may be less effective [55,63]. For a type IV collagen diagnosis, management shifts toward RAS blockade, genotype-appropriate hearing and ocular surveillance, and family screening rather than additional immunosuppression.

Returning to the illustrative vignette, biallelic *NPHS2* variants would argue against prolonged immunosuppression and support kidney-focused surveillance; a *WT1* variant would prompt genotype-informed tumor surveillance and gonadal assessment; whereas biallelic *COQ8B* or another CoQ10-pathway diagnosis would identify a potentially treatable mitochondrial podocytopathy. Thus, the same nephrotic phenotype and FSGS lesion can lead to supportive treatment alone, multisystem tumor surveillance, or targeted metabolic therapy. The principal genotype-specific distinctions and management pathways are summarized in Table 3.

### 6.5. Precision Medicine Take-Home Message

SRNS is a treatment-response phenotype rather than a molecular diagnosis. Genetic testing can distinguish immune-mediated disease from structural, syndromic, or metabolically treatable forms and thereby refine therapy, surveillance, and transplantation counseling.

## 7. The Child with Thrombotic Microangiopathy

### 7.1. Illustrative Clinical Vignette

A 7-year-old girl presents with pallor, fatigue, petechiae, reduced urine output, and hypertension after a nonspecific febrile illness. Laboratory evaluation shows Coombs-negative microangiopathic hemolytic anemia with schistocytes, elevated lactate dehydrogenase, undetectable haptoglobin, thrombocytopenia, and AKI. Urinalysis reveals hematuria and proteinuria. Stool testing does not detect Shiga toxin, and there is no recognized medication, systemic disease, or family history explaining the presentation. The central question is whether this apparently uniform TMA reflects complement dysregulation, severe ADAMTS13 deficiency, an intracellular cobalamin disorder, DGKE-associated disease, or another rare genetic mechanism—diagnoses that require fundamentally different urgent and long-term treatment.

### 7.2. Phenotypic Overlap and Key Genetic Diagnoses

TMA is a clinicopathological syndrome rather than a molecular diagnosis. In children, Shiga toxin–producing *Escherichia coli* hemolytic uremic syndrome (STEC-HUS) is the commonest cause, whereas complement-mediated HUS and other genetic TMAs are much less frequent [64,65]. The shared triad of microangiopathic hemolytic anemia, thrombocytopenia, and organ injury does not reliably identify mechanism; diarrhea may precede complement-mediated disease, complement concentrations may be normal, and extrarenal manifestations overlap.

Complement-mediated TMA most often reflects heterozygous loss-of-function variants in *CFH*, *CFI*, or *CD46*, or gain-of-function variants in *C3* or *CFB*. These variants confer susceptibility with incomplete penetrance rather than inevitable disease, and infection or another endothelial stressor commonly precipitates an episode. Accordingly, a negative family history and normal *C3* do not exclude the diagnosis [66,67,68]. Genomic rearrangements involving *CFH* and the *CFHR* region are also relevant. Anti–factor H autoantibodies, frequently associated with homozygous *CFHR1*/*CFHR3* deletion, represent an acquired but particularly important pediatric complement-mediated subtype.

Biallelic pathogenic variants in *ADAMTS13* cause congenital thrombotic thrombocytopenic purpura (cTTP; Upshaw–Schulman syndrome). Severe ADAMTS13 deficiency, defined by activity below 10%, permits accumulation of ultralarge von Willebrand factor multimers and platelet-rich microthrombi. TTP, historically termed Moschcowitz disease, is exceptionally rare in pediatric practice, and cTTP is rarer still; nevertheless, it must be considered because the diagnosis enables direct enzyme replacement. Onset ranges from the neonatal period to adulthood, and family history may be absent. Marked thrombocytopenia and neurologic involvement can be prominent, although kidney injury does not exclude cTTP. An *ADAMTS13* inhibitor favors immune TTP rather than inherited deficiency [69,70].

The *MMACHC*-related cobalamin C defect is an under-recognized, treatable cause of childhood TMA. Impaired intracellular cobalamin processing produces combined methylmalonic acidemia and homocystinuria, endothelial toxicity, and renal arteriolar and glomerular TMA. Kidney disease may dominate the presentation, while macrocytosis, megaloblastic anemia, neurologic abnormalities, developmental delay, feeding difficulty, failure to thrive, ocular disease, or pulmonary hypertension may be absent or subtle. A normal circulating vitamin B12 concentration does not exclude the disorder. Markedly elevated plasma total homocysteine and plasma or urinary methylmalonic acid, often with low or normal methionine, provide rapid biochemical clues [71,72].

Biallelic *DGKE* variants usually cause recurrent TMA in infancy or early childhood, often with hypertension, persistent proteinuria, hematuria, and later chronic kidney disease. The mechanism involves endothelial, platelet, and podocyte signaling rather than primary complement dysregulation; clinical relapses often become less frequent after early childhood [73,74]. Heterozygous *INF2* variants have been reported in a small number of families with TMA and FSGS, sometimes alongside complement-risk haplotypes. *INF2* should therefore be regarded as a rare pleiotropic association, not a canonical cause equivalent to the established complement genes [75].

### 7.3. Clinical Clues and Genetic Testing Strategy

Evaluation must proceed in parallel because several diagnoses are time-critical. TMA should be confirmed with a complete blood count, blood film, reticulocytes, lactate dehydrogenase, haptoglobin, bilirubin, kidney function, urinalysis, and blood pressure. Stool Shiga toxin PCR or culture should be obtained promptly, while pneumococcal disease and secondary triggers, including severe hypertension, infection, medications, autoimmune disease, malignancy, and transplantation, are assessed [65].

*ADAMTS13* activity and inhibitor testing should be sampled before plasma therapy whenever possible, without delaying emergency treatment. Plasma total homocysteine, methionine, and plasma or urinary methylmalonic acid should be measured early in unexplained pediatric TMA, because biochemical recognition of cobalamin C disease is faster than sequencing and immediately actionable. Complement testing should include C3, C4, factor H, factor I, and anti–factor H antibodies, with soluble C5b-9 where available; however, normal complement results do not exclude complement-mediated TMA [67,76].

Once STEC-HUS and major secondary causes have been evaluated, a comprehensive TMA panel should assess *CFH*, *CFI*, *CD46*, *C3*, *CFB*, *ADAMTS13*, *MMACHC*, and *DGKE*, with validated copy-number and hybrid-gene analysis across the technically complex *CFH*/*CFHR* region. *INF2* is appropriate within a broader kidney-disease panel, particularly when FSGS, persistent proteinuria, neuropathy, or dominant inheritance is present. Trio exome or genome sequencing can follow an unrevealing panel or be used early in atypical multisystem disease. Segregation and expert variant interpretation remain essential; a variant of uncertain significance does not establish the TMA mechanism. Conversely, when complement-mediated disease is strongly suspected, C5 inhibition should not await the genetic result. Acute treatment is therefore guided primarily by the clinical and laboratory suspicion of complement-mediated TMA, while genetic findings assume a greater role in subsequent long-term management.

### 7.4. Genotype-Specific Prognostic and Management Implications

For complement-mediated TMA, prompt terminal complement blockade with eculizumab or long-acting ravulizumab can reverse hematologic activity and improve kidney recovery [77,78]. Both eculizumab and ravulizumab have regulatory indications for pediatric aHUS, although eligibility for ravulizumab is subject to region-specific age and body-weight criteria. Acute C5 inhibition is an established treatment strategy when complement-mediated TMA is strongly suspected clinically and should not depend on prior genetic confirmation. The molecular result is not a prerequisite for acute therapy, but it informs relapse risk, treatment duration, withdrawal surveillance, and transplantation. These longer-term genotype-informed decisions rely on a combination of guideline recommendations, cohort data, and genotype–phenotype associations and should therefore be individualized. Pathogenic variants affecting circulating complement proteins, particularly *CFH*, *CFI*, *C3*, and *CFB*, generally confer greater relapse or graft-recurrence risk than isolated CD46 disease. Evidence informing treatment discontinuation and relapse risk includes mixed pediatric–adult cohorts and should therefore be applied cautiously to individual children. Discontinuation may be considered in selected patients only with rapid access to testing and treatment at relapse [79,80].

In confirmed cTTP, treatment replaces the missing protease rather than blocking complement. Plasma infusion has historically supplied *ADAMTS13*, but recombinant *ADAMTS13* now provides genotype-directed prophylactic and on-demand enzyme replacement. Recombinant ADAMTS13 is currently approved in both the European Union and the United States for the treatment of pediatric and adult patients with congenital TTP. In the phase 3 crossover trial, recombinant therapy restored ADAMTS13 activity to approximately normal levels and produced fewer TTP manifestations than standard plasma-based therapy; the 2025 ISTH update recommends recombinant ADAMTS13 over fresh frozen plasma for prophylaxis in cTTP remission [81,82]. Because the FDA now requires a boxed warning regarding neutralizing antibodies, including serious outcomes and death, clinical response, ADAMTS13 activity, and inhibitor development require close monitoring [83].

For suspected *MMACHC*-related disease, parenteral hydroxocobalamin and betaine should begin as soon as diagnostic samples are obtained, without waiting for molecular confirmation. Folinic acid and carnitine may be added according to metabolic guidance. Prompt treatment can resolve hemolysis, improve kidney function and pulmonary hypertension, and prevent irreversible neurologic injury [71,72]. *DGKE*-associated TMA instead requires supportive kidney care, blood-pressure and proteinuria control, and long-term CKD surveillance. Consistent benefit from complement blockade has not been established; an empiric C5 inhibitor started while the diagnosis is uncertain should be reassessed after molecular confirmation [74]. An INF2 finding directs management toward FSGS/CKD care, neurologic assessment, dominant-family counseling, and cascade testing, but does not itself establish an indication for complement inhibition.

Returning to the illustrative vignette, a pathogenic *CFH* variant would support sustained complement-directed therapy and relapse planning; biallelic ADAMTS13 variants would replace complement blockade with enzyme replacement; biallelic *MMACHC* variants would trigger urgent hydroxocobalamin-based metabolic treatment; whereas biallelic *DGKE* variants would prompt reassessment of prolonged C5 inhibition and emphasize nephroprotection. Thus, the same emergency phenotype can lead to complement inhibition, enzyme replacement, metabolic rescue, or primarily supportive management. The principal genotype-specific distinctions and management pathways are summarized in Table 4.

### 7.5. Precision Medicine Take-Home Message

Childhood TMA is an emergency phenotype with multiple distinct mechanisms. Rapid biochemical, functional, and molecular evaluation can distinguish disorders requiring complement inhibition, enzyme replacement, metabolic therapy, or primarily supportive long-term management. Representative cohort-level diagnostic yields and reported management implications across the four clinical phenotypes are compared in Table 5.

## 8. Cross-Cutting Challenges and Future Directions

### 8.1. Residual Diagnostic Uncertainty and Dynamic Interpretation

The principal unresolved challenge in genomic medicine is determining whether a detected variant explains disease. Broader assays increase the number of variants and candidate genes requiring evaluation, including genes with limited or disputed evidence for disease causation. Clinical conclusions should therefore be restricted to validated relationships between genes and diseases and remain compatible with the relevant molecular mechanism, inheritance pattern, and phenotype [84]. Negative and uncertain findings are provisional because disease gene knowledge, population databases, classification frameworks, and the patient’s phenotype evolve. ACMG guidance distinguishes variant reevaluation from case reanalysis and emphasizes laboratory policies and defined communication pathways when interpretations change [85,86]. The Deciphering Developmental Disorders study illustrates the value of longitudinal analysis, as repeated strategies established diagnoses not recognized during initial assessment [87].

### 8.2. Equitable and Responsible Clinical Implementation

Precision medicine may widen disparities if access to testing, expert interpretation, and targeted treatment depends on geography, ancestry, or financial resources. Underrepresentation in genomic reference datasets increases uncertainty and can produce erroneous classifications. This was demonstrated when variants too common to cause disease in specific ancestry groups were classified as pathogenic because those populations were inadequately represented in reference data [88]. Greater diversity in genomic databases, transparent classification criteria, and responsible data sharing are therefore clinical priorities.

Testing children creates distinctive ethical obligations. Counseling and consent should address uncertain and secondary findings, implications for relatives, data storage, and possible reinterpretation. Assents should be sought when developmentally appropriate, while disclosure should balance present benefit with the child’s developing autonomy [89]. The evolving ACMG list of medically actionable secondary findings reinforces the need to define before testing which findings may be sought and reported [90]. Equitable implementation requires access not only to sequencing, but also to genetic counseling, pediatric nephrology expertise, confirmatory studies, and multidisciplinary interpretation [1,2].

### 8.3. Beyond the Exome: Genome Sequencing and Multiomics

These technologies span markedly different levels of clinical readiness. Genome sequencing is increasingly incorporated into clinical diagnostic pathways, whereas long-read sequencing and clinically directed transcriptomic or functional studies represent emerging tools for selected unresolved cases. Broader multiomic and single-cell approaches and kidney organoids remain predominantly research-stage technologies.

Genome sequencing provides more uniform coverage and can identify structural and deep intronic variants, tandem repeat expansions, and poorly covered coding variants missed by exome sequencing. Its incremental value depends on previous testing. In a large, rare disease cohort, approximately 8% of families required genome sequencing for diagnosis, whereas many diagnoses after a negative exome were recoverable through reanalysis or additional methods, including copy number analysis [91]. A national implementation study likewise showed that diagnostic yield depends on standardized phenotyping, analysis of relatives, validated pipelines, and expert review [92]. Long-read sequencing may resolve complex structural variants, highly homologous regions, and phasing, but cost, throughput, and validation currently favor selective use in unresolved cases [93].

Multiomics can link genomic findings to biological dysfunction, while single-cell RNA sequencing can resolve cellular heterogeneity [94]. In critically ill infants and children, transcriptomics, long-read sequencing, and selected functional assays produced additional diagnoses after rapid genome sequencing [95]. Transcriptomic interpretation is constrained by tissue specificity because an accessible sample may not express the relevant gene or reproduce abnormal splicing [96]. Patient derived cells and kidney organoids may partly address this limitation. In Alport syndrome, kidney organoids generated from induced pluripotent stem cells reproduced differences in collagen IV composition associated with genotype and enabled testing of approaches to correct glomerular basement membrane abnormalities [97]. These models remain complementary and should not independently determine pathogenicity or treatment.

### 8.4. Artificial Intelligence, Therapeutic Translation, and Collaborative Networks

Artificial intelligence may assist phenotype extraction, gene prioritization, variant prediction, and reanalysis, but clinical use requires independent calibration. AlphaMissense, ESM1b, and VARITY can provide clinically relevant evidence at calibrated thresholds, but achieved only modest improvements over previously recommended predictors [98]. Their output should be integrated with population, segregation, functional, and phenotypic evidence rather than treated as an autonomous diagnosis. At present, calibrated computational predictors can support clinical variant interpretation within established classification frameworks, whereas autonomous diagnostic interpretation, treatment recommendation, and broader generative-AI applications remain research-stage or require substantial human oversight. External validation across independent cohorts, laboratories, genes, variant classes, and ancestry groups is essential because performance may not generalize uniformly across clinical settings. Ancestry-related underrepresentation in reference datasets, limited interpretability of complex models, and imperfect calibration may introduce systematic error [88,98]. Accordingly, AI-based pathogenicity predictions should be regarded as one component of the evidence framework and should not independently establish pathogenicity, resolve a variant of uncertain significance, or determine treatment.

The larger objective is to translate molecular diagnosis into effective treatment. RNA-based therapeutics currently span different levels of clinical maturity: RNA interference is already clinically deployed for selected molecularly defined kidney disorders, whereas extension of RNA-based strategies to additional inherited kidney diseases remains a therapeutic frontier. The emergence of RNA interference therapeutics illustrates how molecular understanding can be converted into mechanism-based intervention through selective suppression of disease-relevant gene expression. This therapeutic principle may provide a framework for future RNA-based strategies in other inherited kidney disorders as target selection and delivery technologies improve [6]. Broader clinical translation will require robust natural history data, validated biomarkers, pediatric trial networks, and long-term safety monitoring. Because molecular subgroups are rare, interoperable registries are essential. ERKReg demonstrates how international infrastructure can integrate genetic confirmation, management, treatment performance, and longitudinal outcomes across rare kidney diseases [99]. The future of pediatric precision nephrology lies not in a single technology, but in a learning system connecting rigorous diagnosis, equitable access, functional validation, and mechanism-informed treatment.

## 9. Limitations

This narrative review has limitations. First, although the literature search was structured and sources were cross-verified, the narrative design did not involve a preregistered protocol, duplicate study selection, or systematic data extraction and may therefore be susceptible to selection and publication bias. Second, although the management tables use descriptive evidence-basis categories, no formal risk-of-bias assessment or evidence-grading framework was applied. Consequently, the strength of evidence varies across diagnostic, prognostic, and therapeutic statements. Third, pediatric evidence remains limited for several disorders and genotype-directed interventions, and available studies frequently include small patient populations, necessitating selective inclusion of adult or mixed-age studies when their findings were considered transferable. Fourth, the review focuses on four selected phenotypes and is not intended to provide an exhaustive account of all monogenic kidney diseases or precision-medicine applications in children. Finally, genomic technologies, variant interpretation, gene–disease validity, and targeted therapies are evolving rapidly, and some conclusions may require revision as new evidence emerges. These limitations support cautious, context-dependent interpretation of the clinical implications presented.

## 10. Conclusions

Precision medicine is reshaping pediatric nephrology by demonstrating that shared clinical phenotypes may represent biologically distinct diseases. Persistent microscopic hematuria, bilateral kidney cysts, SRNS, and TMA illustrate a common principle: phenotype provides the diagnostic entry point, whereas molecular diagnosis may refine prognosis, kidney and extrarenal surveillance, treatment, transplantation planning, and family evaluation. Establishing the underlying cause may support early nephroprotection, avoid ineffective immunosuppression, identify tumor or metabolic risks, and, in selected disorders, enable pathway-specific therapy and improve assessment of post-transplant recurrence and inherited risk.

Genomic testing should therefore be embedded within structured phenotyping, pedigree analysis, biochemical and functional studies, and expert variant interpretation. A variant of uncertain significance should not be treated as a definitive diagnosis, while a negative or inconclusive result should prompt review of the phenotype, analytical limitations, potentially missed variant classes, and the need for reanalysis or alternative testing. As genome sequencing, long-read sequencing, multiomics, single-cell approaches, and artificial intelligence advance, clinical integration will require rigorous validation, equitable access, diverse reference datasets, appropriate counseling, and multidisciplinary collaboration.

Ultimately, genotype-informed care does not replace clinical judgment; it strengthens it. Success should be measured not by the number of variants identified, but by whether molecular information improves clinical decisions, avoids harm, and ultimately benefits patients. Linking molecular mechanism to clinical trajectory can transform common presentations into disease specific management pathways and may offer children and their families more accurate, anticipatory, and individualized care.

## Figures and Tables

**Table 1:** Genotype-specific differential diagnosis and management of persistent microscopic hematuria in children.

Genetic Diagnosis	Typical Inheritance	Key Clinical Clues	Extrarenal Manifestations	Genotype-Specific Management Implications	Clinical Risk/Surveillance Intensity	Evidence Basis
*COL4A5*-related XLAS [11,15,18,23]	X-linked; generally more severe in males	Persistent hematuria progressing to albuminuria; greater severity in affected males; compatible maternal family history	Sensorineural hearing loss; anterior lenticonus and other ocular abnormalities	Consider early RAS blockade in affected males; monitor BP, UACR, and eGFR; audiologic and ophthalmologic surveillance; cascade testing	High risk in affected males; intensive kidney and extrarenal surveillance	Guideline/consensus-supported; clinical-trial and observational evidence
Biallelic *COL4A3/COL4A4*–related ARAS [11,15,18,24]	Autosomal recessive	Similar severity in both sexes; consanguinity or apparently sporadic presentation; progression from hematuria to proteinuria and CKD	Hearing and ocular abnormalities may occur	Early nephroprotection with RAS blockade; intensive kidney, hearing, and ocular surveillance; sibling testing	High risk; intensive kidney, audiologic, and ophthalmologic surveillance	Guideline/consensus-supported; observational evidence
Heterozygous *COL4A3/COL4A4*–related disease [11,15,16,17,18]	Autosomal dominant with variable penetrance	Frequently isolated familial hematuria; earlier onset of albuminuria or CKD indicates greater risk	Extrarenal manifestations are uncommon	Lifelong monitoring of BP, UACR, and eGFR; RAS blockade when albuminuria or hypertension develops; reassess unexpectedly severe disease	Variable, generally lower risk than XLAS/ARAS; lifelong risk-adapted monitoring	Guideline/consensus-supported; observational evidence
*CFHR5* nephropathy [19,20,25]	Autosomal dominant with sex-modified severity	Cypriot ancestry; recurrent macroscopic hematuria, often following respiratory infections; higher progression risk in males	Usually absent	Monitor proteinuria, BP, and eGFR; supportive nephroprotection; family screening; complement inhibition is not currently established	Variable, sex-modified risk; closer surveillance with proteinuria or declining kidney function	Observational evidence; emerging evidence for complement inhibition
*MYH9*-related disease [21,22]	Autosomal dominant	Congenital macrothrombocytopenia, giant platelets, bruising, or analyzer-dependent thrombocytopenia accompanying hematuria	Hearing loss, cataracts, and elevated liver enzymes	Hematology involvement; bleeding-risk assessment before biopsy or surgery; avoid inappropriate immunosuppression or splenectomy; kidney and hearing surveillance	Variable kidney and multisystem risk; multidisciplinary surveillance according to phenotype	Observational evidence

ARAS, autosomal recessive Alport syndrome; BP, blood pressure; CKD, chronic kidney disease; eGFR, estimated glomerular filtration rate; RAS, renin–angiotensin system; UACR, urinary albumin-to-creatinine ratio; XLAS, X-linked Alport syndrome. Evidence basis is descriptive and does not constitute a formal risk-of-bias assessment, GRADE rating, or strength-of-recommendation system. Guideline/consensus-supported denotes recommendations from professional guidance or expert consensus; clinical-trial evidence denotes interventional human studies; observational evidence includes cohort, registry, and case-series data; and emerging evidence denotes preliminary, limited, or investigational evidence. More than one category may apply within a row.

**Table 2:** Genotype-specific differential diagnosis and management of bilateral kidney cysts in children.

Genetic Diagnosis	Typical Inheritance	Key Clinical Clues	Extrarenal Manifestations	Genotype-Specific Management Implications	Evidence Basis
*HNF1B*-related disease/17q12 deletion [29,30,31]	Autosomal dominant; frequently de novo	Prenatal hyperechogenicity; cysts, hypoplasia/dysplasia or other CAKUT; hypomagnesemia; hyperuricemia; diabetes	Pancreatic, hepatic and genital tract abnormalities; neurodevelopmental or behavioral features particularly with 17q12 deletion	Monitor BP, UACR, eGFR, magnesium, uric acid, glucose/HbA1c and liver enzymes; assess pancreas and genital tract; neurodevelopmental surveillance for 17q12 deletion; family testing	Observational evidence
*PKHD1*-related ARPKD [32,33,34]	Autosomal recessive	Enlarged hyperechogenic kidneys; poor corticomedullary differentiation; collecting-duct ectasia; early hypertension	Congenital hepatic fibrosis, portal hypertension, splenomegaly, varices and cholangitis	Treat hypertension and CKD complications; lifelong hepatology follow-up; monitor portal-hypertension and cholangitis risk; coordinate kidney–liver transplantation planning; sibling testing	Guideline/consensus-supported; observational evidence
*PKD1/PKD2*-related ADPKD [35,36,37,38]	Autosomal dominant	Bilateral cysts in otherwise preserved parenchyma; affected or previously unrecognized parent; early hypertension; usually greater severity with *PKD1*	Liver cysts and other adult-predominant manifestations; intracranial aneurysm screening is family-history guided	Regular BP, UACR, and eGFR assessment; ambulatory BP monitoring when indicated; established management with RAS blockade for hypertension; individualized imaging and progression-risk counseling; tolvaptan remains a non-established disease-modifying therapy in pediatric ADPKD.	Guideline/consensus-supported; observational evidence; clinical-trial evidence for pediatric tolvaptan, which remains non-established
Monoallelic *IFT140*-related ADPKD-spectrum disease [39,40,41]	Autosomal dominant	Atypical cyst distribution; relatively few large cysts; limited kidney enlargement; childhood presentation possible	Usually kidney-predominant; classic skeletal and retinal ciliopathy features are not expected	Monitor BP, UACR, eGFR and kidney phenotype; provide gene-specific prognostic and reproductive counseling; avoid assuming classic *PKD1* severity or syndromic ciliopathy	Observational and emerging evidence
Biallelic *IFT140*-related ciliopathy [42]	Autosomal recessive	Cystic or tubulointerstitial kidney disease in a syndromic context; possible skeletal clues	Retinal dystrophy and skeletal abnormalities, including Mainzer–Saldino/Jeune-spectrum disease	Multidisciplinary renal, ophthalmologic and skeletal surveillance; developmental assessment when indicated; sibling testing and autosomal-recessive counseling	Observational evidence
*PAX2*-related disorder [43,44,45]	Autosomal dominant; may be de novo	Small dysplastic or hypoplastic kidneys; cysts within CAKUT; proteinuria and progressive CKD	Optic nerve coloboma or dysplasia; possible hearing impairment	Monitor BP, proteinuria and eGFR; formal ophthalmologic examination even without symptoms; consider audiology; parental testing and targeted family assessment	Observational evidence

ADPKD, autosomal dominant polycystic kidney disease; ARPKD, autosomal recessive polycystic kidney disease; BP, blood pressure; CAKUT, congenital anomalies of the kidney and urinary tract; CKD, chronic kidney disease; eGFR, estimated glomerular filtration rate; RAS, renin–angiotensin system; UACR, urinary albumin-to-creatinine ratio. Evidence-basis categories are descriptive and are defined in the note to Table 1.

**Table 3:** Genotype-specific differential diagnosis and management of childhood steroid-resistant nephrotic syndrome.

Genetic Diagnosis	Typical Inheritance and Onset	Key Clinical/Pathological Clues	Extrarenal Manifestations	Genotype-Specific Management Implications	Evidence Basis
*NPHS1*-related disease [50]	Autosomal recessive; usually congenital or infantile, occasionally later	Massive early proteinuria; congenital nephrotic syndrome; childhood SRNS with hypomorphic alleles	Usually kidney-limited	Avoid ineffective immunosuppression; intensive antiproteinuric and nutritional support; transplant planning; counsel regarding rare anti-nephrin antibody–mediated post-transplant recurrence; sibling testing	Guideline/consensus-supported; observational evidence
*NPHS2*-related disease [48,49]	Autosomal recessive; childhood or adolescence	Kidney-limited SRNS; FSGS; consanguinity or affected siblings; variable onset by allelic combination	None typical	Avoid prolonged ineffective immunosuppression; RAS blockade and CKD care; generally low post-transplant recurrence; autosomal-recessive counseling	Guideline/consensus-supported; observational evidence
*WT1*-related disorder [51]	Autosomal dominant, usually de novo; infancy or childhood	DMS or FSGS; early hypertension and CKD; genital findings may be absent	Wilms tumor predisposition; differences in sex development; gonadoblastoma risk	Genotype- and age-adapted tumor surveillance; sex-chromosome and urogenital/gonadal assessment; multidisciplinary counseling; avoid ineffective immunosuppression	Observational evidence
*LAMB2*-related Pierson spectrum [52]	Autosomal recessive; usually congenital or infantile	Congenital nephrotic syndrome or DMS; microcoria may be subtle; later disease with hypomorphic alleles	Ocular, retinal, neurologic, or neuromuscular abnormalities	Supportive kidney care and transplant planning; formal ophthalmology; neurologic and developmental surveillance; sibling testing	Observational evidence
*INF2*-related FSGS [53]	Autosomal dominant; usually adolescence or adulthood	Familial FSGS; progressive proteinuria and CKD; childhood SRNS less typical	Charcot–Marie–Tooth neuropathy in some variants	RAS blockade and CKD care; neurologic examination; autosomal-dominant counseling and cascade testing; avoid assuming immune-mediated FSGS	Observational and emerging evidence, particularly for pediatric SRNS
*COQ8B*/other *CoQ10*-pathway disease [54,55,63]	Autosomal recessive; childhood or adolescence, variable	Insidious proteinuria or SRNS; FSGS; kidney-limited or mitochondrial phenotype	Hearing loss, seizures, developmental regression, ataxia, myopathy, or retinopathy, depending on the gene	Initiate early, sustained CoQ10 supplementation after molecular confirmation; monitor kidney and involved extrarenal systems; avoid ineffective immunosuppression; family testing	Observational evidence for genotype-directed supplementation
*COL4A3*/*COL4A4*/*COL4A5*-related disease [56,57]	X-linked, autosomal dominant, or autosomal recessive; variable onset	Proteinuria and/or FSGS; hematuria and characteristic GBM changes may be subtle or absent initially	Sensorineural hearing loss and ocular abnormalities may be absent or emerge later	RAS blockade; genotype-appropriate hearing and ocular surveillance; family screening; reinterpret FSGS as secondary injury; avoid ineffective immunosuppression	Guideline/consensus-supported; observational evidence

CKD, chronic kidney disease; DMS, diffuse mesangial sclerosis; eGFR, estimated glomerular filtration rate; FSGS, focal segmental glomerulosclerosis; GBM, glomerular basement membrane; RAS, renin–angiotensin system; SRNS, steroid-resistant nephrotic syndrome. Evidence-basis categories are descriptive and are defined in the note to Table 1.

**Table 4:** Genotype-specific differential diagnosis and management of childhood thrombotic microangiopathy.

Genetic Diagnosis	Typical Inheritance and Onset	Key Clinical/Biochemical Clues	Extrarenal Manifestations	Genotype-Specific Management Implications	Evidence Basis
Complement-mediated TMA: *CFH*, *CFI*, *CD46*, *C3*, *CFB* [66,67,68]	Usually autosomal dominant susceptibility with incomplete penetrance; any childhood age	AKI-predominant TMA; common trigger; C3 may be normal; family history may be absent; anti–factor H autoantibodies, often associated with homozygous CFHR1–CFHR3 deletion	Neurologic, cardiac, gastrointestinal, pulmonary, or ocular involvement	Prompt eculizumab or ravulizumab; vaccination/infection prophylaxis; genotype-informed treatment duration, relapse surveillance, transplant planning, and family testing; immunosuppression and antibody-titer monitoring for anti–factor H antibody–associated disease	Guideline/consensus-supported; clinical-trial and observational evidence
*ADAMTS13*-related cTTP [69,81,82]	Autosomal recessive; neonatal period to adulthood; exceptionally rare in children	ADAMTS13 activity < 10% without inhibitor; severe thrombocytopenia; recurrent episodes	Neurologic and cardiac ischemia; renal involvement variable	Recombinant ADAMTS13 preferred for prophylaxis and available for on-demand replacement; plasma-derived replacement if unavailable; monitor activity and inhibitors	Guideline-supported; phase 3 clinical-trial evidence
*MMACHC*-related cobalamin C disease [71,72]	Autosomal recessive; infancy to adolescence	Very high total homocysteine and methylmalonic acid; low-to-normal methionine; normal serum B12 possible	Neurodevelopmental, ocular, feeding, cardiopulmonary, or pulmonary-hypertension phenotype	Urgent parenteral hydroxocobalamin plus betaine; metabolic follow-up; do not await sequencing before treatment	Observational evidence
*DGKE*-associated TMA/nephropathy [73,74]	Autosomal recessive; usually infancy or early childhood	Recurrent TMA; hypertension; persistent proteinuria/hematuria; progressive CKD	Usually kidney-predominant	Supportive CKD and antiproteinuric care; no established consistent benefit from C5 blockade; reassess empiric complement inhibition	Observational evidence
*INF2*-associated TMA/FSGS [75]	Autosomal dominant; variable, often later kidney disease	Rare association; FSGS, proteinuria, CKD; possible coexisting complement-risk variants	Charcot–Marie–Tooth neuropathy in some variants	FSGS/CKD care; neurologic assessment; dominant-family counseling and cascade testing; not a stand-alone indication for complement blockade	Emerging evidence based on limited observational reports

AKI, acute kidney injury; cTTP, congenital thrombotic thrombocytopenic purpura; CKD, chronic kidney disease; FSGS, focal segmental glomerulosclerosis; TMA, thrombotic microangiopathy. Evidence-basis categories are descriptive and are defined in the note to Table 1.

**Table 5:** Representative cohort-level molecular diagnostic yield and reported management impact across the four clinical phenotypes.

Phenotype	Representative Cohort	Population and Testing Strategy	Reported Molecular or Etiologic Yield	Principal Genes or Findings	Reported Management Impact
Persistent microscopic hematuria	Ng et al., 2025 [12]	Children and young people aged <18 years with persistent microscopic hematuria; 134 underwent targeted sequencing	91/134 (67.9%)	*COL4A3*, *COL4A4*, and *COL4A5*; *COL4A5* was the most frequently affected gene	Numerical proportion altered: NR. Molecular classification supports Alport subtype-specific prognosis, RAS-blockade decisions, hearing and ocular surveillance, and cascade testing [11,12,15].
Bilateral cystic or hyperechogenic kidneys	Bracciamà et al., 2025 [28]	Pediatric cohort with prenatal or childhood ultrasonographic abnormalities; clinical exome sequencing with staged cystic-kidney gene analysis; *n* = 70	53/70 (75.7%)	Principally *PKD1*, *PKHD1*, *HNF1B*, and *PKD2*, with additional cystic-disease genes	Numerical proportion altered: NR. Molecular diagnosis distinguishes ADPKD, ARPKD, HNF1B/17q12-related disease, and other ciliopathies, redirecting kidney and extrarenal surveillance and recurrence-risk counseling [28,29,30,31,32,33,34,36,37,42,43,44,45].
Steroid-resistant nephrotic syndrome	Bierzynska et al., 2017 [49]	National pediatric UK cohort; whole-exome sequencing focused on 53 nephrotic-syndrome genes; *n* = 187	49/187 (26.2%) overall; 30.8% in primary SRNS and 0% in secondary SRNS	Most frequently *NPHS1*, *NPHS2*, and *WT1*, with disease-causing variants in 14 additional genes	Numerical proportion altered: NR. Testing stratified monogenic from nonmonogenic disease, informed avoidance of prolonged ineffective immunosuppression, gene-specific surveillance or treatment, and post-transplant recurrence counseling [47,49,51,55,58,59,60,61,62,63].
Thrombotic microangiopathy, represented by an aHUS cohort	Noris et al., 2010 [66]	International registry of 273 familial and sporadic aHUS patients; mixed-age, study-era targeted complement analysis	Complement-gene mutations in 131/273 (48.0%); mutations and/or anti-CFH autoantibodies in 139/273 (50.9%)	*CFH*, *CFI*, *CD46* (*MCP*), *C3*, and *THBD*, with rarer or combined abnormalities	Numerical proportion altered: NR. Molecular findings predicted prognosis, plasma response, relapse, and graft recurrence and now contribute to complement-inhibitor, withdrawal-surveillance, and transplantation planning [66,67,74,76,77,78,79,80].
Cross-phenotype renal genetics cohort, contextual benchmark	Jayasinghe et al., 2021 [9]	Prospective mixed pediatric–adult renal genetics cohort; clinically accredited exome sequencing; *n* = 204	80/204 (39.2%)	Thirty-five distinct genetic disorders	Management influenced in 47/80 molecularly diagnosed patients (58.8%; 23.0% of all tested), including altered surveillance in 35/80, altered treatment in 16/80, and avoidance of kidney biopsy in 10/80.

ADPKD, autosomal dominant polycystic kidney disease; aHUS, atypical hemolytic uremic syndrome; ARPKD, autosomal recessive polycystic kidney disease; NR, not reported; RAS, renin–angiotensin system; SRNS, steroid-resistant nephrotic syndrome. Diagnostic yield represents the author-reported detection of pathogenic, likely pathogenic, disease-causing, or causative variants according to each study’s definitions. For Noris et al. [66], the higher etiologic yield includes anti-CFH autoantibodies and is therefore not a purely genetic diagnostic yield. That cohort included children and adults with aHUS and should not be considered representative of unselected pediatric TMA. Estimates should not be compared directly because cohort selection, phenotype definitions, testing platforms, and variant-classification methods differed.

## Data Availability

No new data were created or analyzed in this study. Data sharing is not applicable to this article.

## Abbreviations

The following abbreviations are used in this manuscript:

ACMG	American College of Medical Genetics and Genomics
ADPKD	Autosomal dominant polycystic kidney disease
AKI	Acute kidney injury
AMP	Association for Molecular Pathology
ARAS	Autosomal recessive Alport syndrome
ARPKD	Autosomal recessive polycystic kidney disease
BP	Blood pressure
CAKUT	Congenital anomalies of the kidney and urinary tract
CFH	Complement Factor H
CKD	Chronic kidney disease
CNV	Copy-number variant
CoQ10	Coenzyme Q10
cTTP	Congenital thrombotic thrombocytopenic purpura
DMS	Diffuse mesangial sclerosis
DNA	Deoxyribonucleic acid
eGFR	Estimated glomerular filtration rate
ERKReg	European Rare Kidney Disease Registry
FSGS	Focal segmental glomerulosclerosis
GBM	Glomerular basement membrane
HbA1c	Glycated hemoglobin A1c
HNF1B	Hepatocyte Nuclear Factor 1-Beta
HUS	Hemolytic uremic syndrome
ISTH	International Society on Thrombosis and Haemostasis
KDIGO	Kidney Disease: Improving Global Outcomes
MEDLINE	Medical Literature Analysis and Retrieval System Online
NS	Nephrotic syndrome
PCR	Polymerase chain reaction
RAS	Renin–angiotensin system
RNA	Ribonucleic acid
SRNS	Steroid-resistant nephrotic syndrome
STEC	Shiga toxin-producing *Escherichia coli*
STEC-HUS	Shiga toxin-producing *Escherichia coli*-associated hemolytic uremic syndrome
SV	Structural variant
TMA	Thrombotic microangiopathy
TTP	Thrombotic thrombocytopenic purpura
UACR	Urinary albumin-to-creatinine ratio
XLAS	X-linked Alport syndrome
WES	Whole-exome sequencing
WGS	Whole-genome sequencing

## References

[1] Knoers N., Antignac C., Bergmann C., Dahan K., Giglio S., Heidet L., Lipska-Ziętkiewicz B.S., Noris M., Remuzzi G., Vargas-Poussou R. (2022). Genetic Testing in the Diagnosis of Chronic Kidney Disease: Recommendations for Clinical Practice. Nephrol. Dial. Transplant..

[2] Aron A.W., Dahl N.K. (2024). Clinical Genetic Testing in Nephrology: Core Curriculum 2024. Am. J. Kidney Dis..

[3] Rao J., Liu X., Mao J., Tang X., Shen Q., Li G., Sun L., Bi Y., Wang X., Qian Y. (2019). Genetic Spectrum of Renal Disease for 1001 Chinese Children Based on a Multicenter Registration System. Clin. Genet..

[4] Groopman E.E., Marasa M., Cameron-Christie S., Petrovski S., Aggarwal V.S., Milo-Rasouly H., Li Y., Zhang J., Nestor J., Krithivasan P. (2019). Diagnostic Utility of Exome Sequencing for Kidney Disease. N. Engl. J. Med..

[5] Mann N., Braun D.A., Amann K., Tan W., Shril S., Connaughton D.M., Nakayama M., Schneider R., Kitzler T.M., van der Ven A.T. (2019). Whole-Exome Sequencing Enables a Precision Medicine Approach for Kidney Transplant Recipients. J. Am. Soc. Nephrol..

[6] Dotis J., Fourikou M. (2026). The Dawn of Precision Medicine in Pediatric Nephrology: Lumasiran and the Era of siRNA Therapies for Primary Hyperoxaluria Type 1. J. Pers. Med..

[7] Köhler S., Gargano M., Matentzoglu N., Carmody L.C., Lewis-Smith D., Vasilevsky N.A., Danis D., Balagura G., Baynam G., Brower A.M. (2021). Human Phenotype Ontology in 2021. Nucleic Acids Res..

[8] Richards S., Aziz N., Bale S., Bick D., Das S., Gastier-Foster J., Grody W.W., Hegde M., Lyon E., Spector E. (2015). Standards and guidelines for the interpretation of sequence variants: A joint consensus recommendation of the American College of Medical Genetics and Genomics and the Association for Molecular Pathology. Genet. Med..

[9] Jayasinghe K., Stark Z., Kerr P.G., Gaff C., Martyn M., Whitlam J., Creighton B., Donaldson E., Hunter M., Jarmolowicz A. (2021). Clinical impact of genomic testing in patients with suspected monogenic kidney disease. Genet. Med..

[10] Latta K., Boeckhaus J., Weinreich I., Borisch A., Müller D., Kaufmann S., Mentzel H.-J., Vauth F., Göbel H., Furtwängler R. (2026). German Clinical Practice Guideline on Microhematuria in Children and Young Adults: Evaluating Early Detection of Kidney Disease. Kidney Int. Rep..

[11] Torra R., Lipska-Ziętkiewicz B., Acke F., Antignac C., Becker J.U., Cornec-Le Gall E., van Eerde A.M., Feltgen N., Ferrari R., Gale D.P. (2025). Diagnosis, management and treatment of the Alport syndrome—2024 guideline on behalf of ERKNet, ERA and ESPN. Nephrol. Dial. Transplant..

[12] Ng N.S.L., Yamamura T., Shenoy M., Stuart H.M., Lennon R. (2025). Detection of Alport gene variants in children and young people with persistent haematuria. Pediatr. Nephrol..

[13] Alge J.L., Bekheirnia N., Willcockson A.R., Qin X., Scherer S.E., Braun M.C., Bekheirnia M.R. (2023). Variants in genes coding for collagen type IV α-chains are frequent causes of persistent, isolated hematuria during childhood. Pediatr. Nephrol..

[14] Rheault M.N., McLaughlin H.M., Mitchell A., Blake L.E., Devarajan P., Warady B.A., Gibson K.L., Lieberman K.V. (2023). COL4A gene variants are common in children with hematuria and a family history of kidney disease. Pediatr. Nephrol..

[15] Savige J., Lipska-Ziętkiewicz B.S., Watson E., Hertz J.M., Deltas C., Mari F., Hilbert P., Plevova P., Byers P., Cerkauskaite A. (2022). Guidelines for genetic testing and management of Alport syndrome. Clin. J. Am. Soc. Nephrol..

[16] Hu N., Sun L., Dai X., Zhao Z., Zhang X., Rao J., Zhou X., Fang Y., Shi Y., Jin S. (2025). Age at disease onset and risk of chronic kidney disease in patients with heterozygous disease-causing variants in COL4A3 and COL4A4. Clin. Kidney J..

[17] Mastrangelo A., Madeira C., Castorina P., Giani M., Montini G. (2022). Heterozygous COL4A3/COL4A4 mutations: The hidden part of the iceberg?. Nephrol. Dial. Transplant..

[18] Dotis J. (2026). Redefining Alport syndrome in the era of genomic medicine: Time for a unified, genotype-driven nomenclature. Pediatr. Nephrol..

[19] Gale D.P., de Jorge E.G., Cook H.T., Martinez-Barricarte R., Hadjisavvas A., McLean A.G., Pusey C.D., Pierides A., Kyriacou K., Athanasiou Y. (2010). Identification of a mutation in complement factor H-related protein 5 in patients of Cypriot origin with glomerulonephritis. Lancet.

[20] Athanasiou Y., Voskarides K., Gale D.P., Damianou L., Patsias C., Zavros M., Maxwell P.H., Cook H.T., Demosthenous P., Hadjisavvas A. (2011). Familial C3 glomerulopathy associated with CFHR5 mutations: Clinical characteristics of 91 patients in 16 pedigrees. Clin. J. Am. Soc. Nephrol..

[21] Tabibzadeh N., Fleury D., Labatut D., Bridoux F., Lionet A., Jourde-Chiche N., Vrtovsnik F., Schlegel N., Vanhille P. (2019). MYH9-related disorders display heterogeneous kidney involvement and outcome. Clin. Kidney J..

[22] Savoia A., Pecci A., Adam M.P., Bick S., Mirzaa G.M., Wallace S.E., Amemiya A. (2008). MYH9-Related Disease. GeneReviews ®.

[23] Gross O., Tönshoff B., Weber L.T., Pape L., Latta K., Fehrenbach H., Lange-Sperandio B., Zappel H., Hoyer P., Staude H. (2020). A multicenter, randomized, placebo-controlled, double-blind phase 3 trial with open-arm comparison indicates safety and efficacy of nephroprotective therapy with ramipril in children with Alport’s syndrome. Kidney Int..

[24] Zhang Y., Böckhaus J., Wang F., Wang S., Rubel D., Gross O., Ding J. (2021). Genotype–phenotype correlations and nephroprotective effects of RAAS inhibition in patients with autosomal recessive Alport syndrome. Pediatr. Nephrol..

[25] Apetrii M., Costache A.D., Costache Enache I.I., Voroneanu L., Covic A.S., Kanbay M., Covic A. (2025). Complement system inhibitors in nephrology: An update—Narrative review. Int. J. Mol. Sci..

[26] Gimpel C., Avni F.E., Bergmann C., Cetiner M., Habbig S., Haffner D., König J., Konrad M., Liebau M.C., Pape L. (2018). Perinatal Diagnosis, Management, and Follow-up of Cystic Renal Diseases: A Clinical Practice Recommendation With Systematic Literature Reviews. JAMA Pediatr..

[27] Gimpel C., Avni E.F., Breysem L., Burgmaier K., Caroli A., Cetiner M., Haffner D., Hartung E.A., Franke D., König J. (2019). Imaging of Kidney Cysts and Cystic Kidney Diseases in Children: An International Working Group Consensus Statement. Radiology.

[28] Bracciamà V., Vaisitti T., Mioli F., Faini A.C., Del Prever G.M.B., Martins V.H., Camilla R., Mattozzi F., Pieretti S., Luca M. (2025). Matching Clinical and Genetic Data in Pediatric Patients at Risk of Developing Cystic Kidney Disease. Pediatr. Nephrol..

[29] Clissold R.L., Hamilton A.J., Hattersley A.T., Ellard S., Bingham C. (2015). HNF1B-Associated Renal and Extra-Renal Disease—An Expanding Clinical Spectrum. Nat. Rev. Nephrol..

[30] Adalat S., Woolf A.S., Johnstone K.A., Wirsing A., Harries L.W., Long D.A., Hennekam R.C., Ledermann S.E., Rees L., van’t Hoff W. (2009). HNF1B Mutations Associate with Hypomagnesemia and Renal Magnesium Wasting. J. Am. Soc. Nephrol..

[31] Buffin-Meyer B., Richard J., Guigonis V., Weber S., König J., Heidet L., Moussaoui N., Vu J.-P., Faguer S., Casemayou A. (2024). Renal and Extrarenal Phenotypes in Patients With HNF1B Variants and Chromosome 17q12 Microdeletions. Kidney Int. Rep..

[32] Bergmann C., Senderek J., Windelen E., Küpper F., Middeldorf I., Schneider F., Dornia C., Rudnik-Schöneborn S., Konrad M., Schmitt C.P. (2005). Clinical Consequences of PKHD1 Mutations in 164 Patients with Autosomal-Recessive Polycystic Kidney Disease (ARPKD). Kidney Int..

[33] Guay-Woodford L.M., Bissler J.J., Braun M.C., Bockenhauer D., Cadnapaphornchai M.A., Dell K.M., Kerecuk L., Liebau M.C., Alonso-Peclet M.H., Shneider B. (2014). Expert Recommendations for the Diagnosis and Management of Autosomal Recessive Polycystic Kidney Disease: Report of an International Conference. J. Pediatr..

[34] Burgmaier K., Broekaert I.J., Liebau M.C. (2023). Autosomal Recessive Polycystic Kidney Disease: Diagnosis, Prognosis, and Management. Adv. Kidney Dis. Health.

[35] Bergmann C., von Bothmer J., Ortiz Brüchle N., Venghaus A., Frank V., Fehrenbach H., Hampel T., Pape L., Buske A., Jonsson J. (2011). Mutations in Multiple PKD Genes May Explain Early and Severe Polycystic Kidney Disease. J. Am. Soc. Nephrol..

[36] Gimpel C., Bergmann C., Bockenhauer D., Breysem L., Cadnapaphornchai M.A., Cetiner M., Dudley J., Emma F., Konrad M., Harris T. (2019). International Consensus Statement on the Diagnosis and Management of Autosomal Dominant Polycystic Kidney Disease in Children and Young People. Nat. Rev. Nephrol..

[37] (2025). Kidney Disease: Improving Global Outcomes (KDIGO) ADPKD Work Group. KDIGO 2025 Clinical Practice Guideline for the Evaluation, Management, and Treatment of Autosomal Dominant Polycystic Kidney Disease (ADPKD). Kidney Int..

[38] Mekahli D., Guay-Woodford L.M., Cadnapaphornchai M.A., Greenbaum L.A., Litwin M., Seeman T., Dandurand A., Shi L., Sikes K., Shoaf S.E. (2023). Tolvaptan for Children and Adolescents with Autosomal Dominant Polycystic Kidney Disease: Randomized Controlled Trial. Clin. J. Am. Soc. Nephrol..

[39] Senum S.R., Li Y.S.M., Benson K.A., Joli G., Olinger E., Lavu S., Madsen C.D., Gregory A.V., Neatu R., Kline T.L. (2022). Monoallelic IFT140 Pathogenic Variants Are an Important Cause of the Autosomal Dominant Polycystic Kidney-Spectrum Phenotype. Am. J. Hum. Genet..

[40] Zagorec N., Calamel A., Delaporte M., Olinger E., Orr S., Sayer J.A., Pillay V.-G., Denommé-Pichon A.-S., Tran Mau-Them F., Nambot S. (2025). Clinical Spectrum and Prognosis of Atypical Autosomal Dominant Polycystic Kidney Disease Caused by Monoallelic Pathogenic Variants of IFT140. Am. J. Kidney Dis..

[41] Griffiths J.D., Ehidiamhen G., Lopez-Garcia S.C., Ong A.C.M. (2026). Monoallelic IFT140 Variants Causing Childhood-Onset Autosomal Dominant Polycystic Kidney Disease. Am. J. Kidney Dis..

[42] Perrault I., Saunier S., Hanein S., Filhol E., Bizet A.A., Collins F., Salih M.A.M., Gerber S., Delphin N., Bigot K. (2012). Mainzer–Saldino Syndrome Is a Ciliopathy Caused by IFT140 Mutations. Am. J. Hum. Genet..

[43] Thomas R., Sanna-Cherchi S., Warady B.A., Furth S.L., Kaskel F.J., Gharavi A.G. (2011). HNF1B and PAX2 Mutations Are a Common Cause of Renal Hypodysplasia in the CKiD Cohort. Pediatr. Nephrol..

[44] Rossanti R., Morisada N., Nozu K., Kamei K., Horinouchi T., Yamamura T., Minamikawa S., Fujimura J., Nagano C., Sakakibara N. (2020). Clinical and Genetic Variability of PAX2-Related Disorder in the Japanese Population. J. Hum. Genet..

[45] Kim J.H., Ahn Y.H., Jang Y., Park E., Lee H., Kim S.H., Song J.Y., Han K.H., Jung J., Lee J.H. (2025). Genotype of PAX2-Related Disorders Correlates with Kidney and Ocular Manifestations. Eur. J. Hum. Genet..

[46] Sadowski C.E., Lovric S., Ashraf S., Pabst W.L., Gee H.Y., Kohl S., Engelmann S., Vega-Warner V., Fang H., Halbritter J. (2015). A Single-Gene Cause in 29.5% of Cases of Steroid-Resistant Nephrotic Syndrome. J. Am. Soc. Nephrol..

[47] Trautmann A., Vivarelli M., Samuel S., Gipson D., Sinha A., Schaefer F., Hui N.K., Boyer O., Saleem M.A., Feltran L. (2020). IPNA Clinical Practice Recommendations for the Diagnosis and Management of Children with Steroid-Resistant Nephrotic Syndrome. Pediatr. Nephrol..

[48] Trautmann A., Bodria M., Ozaltin F., Gheisari A., Melk A., Azocar M., Anarat A., Caliskan S., Emma F., Gellermann J. (2015). Spectrum of Steroid-Resistant and Congenital Nephrotic Syndrome in Children: The PodoNet Registry Cohort. Clin. J. Am. Soc. Nephrol..

[49] Bierzynska A., McCarthy H.J., Soderquest K., Sen E.S., Colby E., Ding W.Y., Nabhan M.M., Kerecuk L., Hegde S., Hughes D. (2017). Genomic and Clinical Profiling of a National Nephrotic Syndrome Cohort Advocates a Precision Medicine Approach to Disease Management. Kidney Int..

[50] Lipska-Ziętkiewicz B.S., Ozaltin F., Hölttä T., Bockenhauer D., Bérody S., Levtchenko E., Vivarelli M., Webb H., Haffner D., Schaefer F. (2020). Genetic Aspects of Congenital Nephrotic Syndrome: A Consensus Statement from the ERKNet–ESPN Inherited Glomerulopathy Working Group. Eur. J. Hum. Genet..

[51] Lipska B.S., Ranchin B., Iatropoulos P., Gellermann J., Melk A., Ozaltin F., Caridi G., Seeman T., Tory K., Jankauskiene A. (2014). Genotype–Phenotype Associations in WT1 Glomerulopathy. Kidney Int..

[52] Choi H.J., Lee B.H., Kang J.H., Jeong H.J., Moon K.C., Ha I.S., Yu Y.S., Matejas V., Zenker M., Choi Y. (2008). Variable Phenotype of Pierson Syndrome. Pediatr. Nephrol..

[53] Barua M., Brown E.J., Charoonratana V.T., Genovese G., Sun H., Pollak M.R. (2013). Mutations in the INF2 Gene Account for a Significant Proportion of Familial but Not Sporadic Focal and Segmental Glomerulosclerosis. Kidney Int..

[54] Ashraf S., Gee H.Y., Woerner S., Xie L.X., Vega-Warner V., Lovric S., Fang H., Song X., Cattran D.C., Avila-Casado C. (2013). ADCK4 Mutations Promote Steroid-Resistant Nephrotic Syndrome through CoQ10 Biosynthesis Disruption. J. Clin. Investig..

[55] Tan W., Airik R. (2021). Primary Coenzyme Q10 Nephropathy, a Potentially Treatable Form of Steroid-Resistant Nephrotic Syndrome. Pediatr. Nephrol..

[56] Quinlan C., Rheault M.N. (2021). Genetic Basis of Type IV Collagen Disorders of the Kidney. Clin. J. Am. Soc. Nephrol..

[57] Dotis J., Kondou A., Liapis G., Ververi A., Kollios K., Printza N. (2026). When Genes Reveal the Truth: Alport Syndrome Mimicking Steroid-Resistant Nephrotic Syndrome. Pediatr. Rep..

[58] Santín S., Bullich G., Tazón-Vega B., García-Maset R., Giménez I., Silva I., Ruíz P., Ballarín J., Torra R., Ars E. (2011). Clinical Utility of Genetic Testing in Children and Adults with Steroid-Resistant Nephrotic Syndrome. Clin. J. Am. Soc. Nephrol..

[59] Preston R., Stuart H.M., Lennon R. (2019). Genetic Testing in Steroid-Resistant Nephrotic Syndrome: Why, Who, When and How?. Pediatr. Nephrol..

[60] Cheong H.I. (2020). Genetic Tests in Children with Steroid-Resistant Nephrotic Syndrome. Kidney Res. Clin. Pract..

[61] Lovric S., Ashraf S., Tan W., Hildebrandt F. (2016). Genetic Testing in Steroid-Resistant Nephrotic Syndrome: When and How?. Nephrol. Dial. Transplant..

[62] Büscher A.K., Beck B.B., Melk A., Hoefele J., Kranz B., Bamborschke D., Baig S., Lange-Sperandio B., Jungraithmayr T., Weber L.T. (2016). Rapid Response to Cyclosporin A and Favorable Renal Outcome in Nongenetic versus Genetic Steroid-Resistant Nephrotic Syndrome. Clin. J. Am. Soc. Nephrol..

[63] Drovandi S., Lipska-Ziętkiewicz B.S., Ozaltin F., Emma F., Gulhan B., Boyer O., Trautmann A., Xu H., Shen Q., Rao J. (2022). Oral Coenzyme Q10 Supplementation Leads to Better Preservation of Kidney Function in Steroid-Resistant Nephrotic Syndrome Due to Primary Coenzyme Q10 Deficiency. Kidney Int..

[64] Loirat C., Fakhouri F., Ariceta G., Besbas N., Bitzan M., Bjerre A., Coppo R., Emma F., Johnson S., Karpman D. (2016). An International Consensus Approach to the Management of Atypical Hemolytic Uremic Syndrome in Children. Pediatr. Nephrol..

[65] Palma L.M.P., Vaisbich-Guimarães M.H., Sridharan M., Tran C.L., Sethi S. (2022). Thrombotic Microangiopathy in Children. Pediatr. Nephrol..

[66] Noris M., Caprioli J., Bresin E., Mossali C., Pianetti G., Gamba S., Daina E., Fenili C., Castelletti F., Sorosina A. (2010). Relative Role of Genetic Complement Abnormalities in Sporadic and Familial aHUS and Their Impact on Clinical Phenotype. Clin. J. Am. Soc. Nephrol..

[67] Goodship T.H.J., Cook H.T., Fakhouri F., Fervenza F.C., Frémeaux-Bacchi V., Kavanagh D., Nester C.M., Noris M., Pickering M.C., Rodríguez de Córdoba S. (2017). Atypical Hemolytic Uremic Syndrome and C3 Glomerulopathy: Conclusions from a “Kidney Disease: Improving Global Outcomes” (KDIGO) Controversies Conference. Kidney Int..

[68] Bogdan R.-G., Anderco P., Ichim C., Cimpean A.-M., Todor S.B., Glaja-Iliescu M., Crainiceanu Z.P., Popa M.L. (2025). Atypical Hemolytic Uremic Syndrome: A Review of Complement Dysregulation, Genetic Susceptibility and Multiorgan Involvement. J. Clin. Med..

[69] Alwan F., Vendramin C., Liesner R., Clark A., Lester W., Dutt T., Thomas W., Gooding R., Biss T., Watson H.G. (2019). Characterization and Treatment of Congenital Thrombotic Thrombocytopenic Purpura. Blood.

[70] van Dorland H.A., Mansouri Taleghani M., Sakai K., Friedman K.D., George J.N., Hrachovinova I., Knöbl P.N., von Krogh A.S., Schneppenheim R., Aebi-Huber I. (2019). The International Hereditary Thrombotic Thrombocytopenic Purpura Registry: Key Findings at Enrollment until 2017. Haematologica.

[71] Beck B.B., van Spronsen F.J., Diepstra A., Berger R.M.F., Kömhoff M. (2017). Renal Thrombotic Microangiopathy in Patients with cblC Defect: Review of an Under-Recognized Entity. Pediatr. Nephrol..

[72] Lemoine M., François A., Grangé S., Rabant M., Châtelet V., Cassiman D., Cornec-Le Gall E., Ambrosetti D., Deschênes G., Benoist J.-F. (2018). Cobalamin C Deficiency Induces a Typical Histopathological Pattern of Renal Arteriolar and Glomerular Thrombotic Microangiopathy. Kidney Int. Rep..

[73] Lemaire M., Frémeaux-Bacchi V., Schaefer F., Choi M., Tang W.H., Le Quintrec M., Fakhouri F., Taque S., Nobili F., Martinez F. (2013). Recessive Mutations in DGKE Cause Atypical Hemolytic-Uremic Syndrome. Nat. Genet..

[74] Brocklebank V., Kumar G., Howie A.J., Chandar J., Milford D.V., Craze J., Evans J., Finlay E., Freundlich M., Gale D.P. (2020). Long-Term Outcomes and Response to Treatment in Diacylglycerol Kinase Epsilon Nephropathy. Kidney Int..

[75] Challis R.C., Ring T., Xu Y., Wong E.K.S., Flossmann O., Roberts I.S.D., Ahmed S., Wetherall M., Salkus G., Brocklebank V. (2017). Thrombotic Microangiopathy in Inverted Formin 2–Mediated Renal Disease. J. Am. Soc. Nephrol..

[76] Doreille A., Rafat C., Rondeau E., Mesnard L. (2023). How I Treat Thrombotic Microangiopathy in the Era of Rapid Genomics. Blood.

[77] Greenbaum L.A., Fila M., Ardissino G., Al-Akash S.I., Evans J., Henning P., Lieberman K.V., Maringhini S., Pape L., Rees L. (2016). Eculizumab Is a Safe and Effective Treatment in Pediatric Patients with Atypical Hemolytic Uremic Syndrome. Kidney Int..

[78] Ariceta G., Dixon B.P., Kim S.H., Kapur G., Mauch T., Ortiz S., Vallee M., Denker A.E., Kang H.G., Greenbaum L.A. (2021). The Long-Acting C5 Inhibitor, Ravulizumab, Is Effective and Safe in Pediatric Patients with Atypical Hemolytic Uremic Syndrome Naïve to Complement Inhibitor Treatment. Kidney Int..

[79] Fakhouri F., Fila M., Hummel A., Ribes D., Sellier-Leclerc A.-L., Ville S., Pouteil-Noble C., Coindre J.-P., Le Quintrec M., Rondeau E. (2021). Eculizumab Discontinuation in Children and Adults with Atypical Hemolytic-Uremic Syndrome: A Prospective Multicenter Study. Blood.

[80] Brocklebank V., Walsh P.R., Smith-Jackson K., Hallam T.M., Marchbank K.J., Wilson V., Bigirumurame T., Dutt T., Montgomery E.K., Malina M. (2023). Atypical Hemolytic Uremic Syndrome in the Era of Terminal Complement Inhibition: An Observational Cohort Study. Blood.

[81] Scully M., Antun A., Cataland S.R., Coppo P., Dossier C., Biebuyck N., Hassenpflug W.A., Kentouche K., Knöbl P., Kremer Hovinga J.A. (2024). Recombinant ADAMTS13 in Congenital Thrombotic Thrombocytopenic Purpura. N. Engl. J. Med..

[82] Zheng X.L., Al-Housni Z., Cataland S.R., Coppo P., Geldziler B., Germini F., Iorio A., Keepanasseril A., Masias C., Matsumoto M. (2025). 2025 Focused Update of the 2020 ISTH Guidelines for Management of Thrombotic Thrombocytopenic Purpura. J. Thromb. Haemost..

[83] U.S. Food and Drug Administration (2026). Safety Labeling Change Order and Safety Labeling Change Notification Letter—ADZYNMA (ADAMTS13, Recombinant-krhn). https://www.fda.gov/media/192821/download.

[84] Strande N.T., Riggs E.R., Buchanan A.H., Ceyhan-Birsoy O., DiStefano M., Dwight S.S., Goldstein J., Ghosh R., Seifert B.A., Sneddon T.P. (2017). Evaluating the Clinical Validity of Gene-Disease Associations: An Evidence-Based Framework Developed by the Clinical Genome Resource. Am. J. Hum. Genet..

[85] David K.L., Best R.G., Brenman L.M., Bush L., Deignan J.L., Flannery D., Hoffman J.D., Holm I., Miller D.T., O’Leary J. (2019). Patient re-contact after revision of genomic test results: Points to consider—A statement of the American College of Medical Genetics and Genomics. Genet. Med..

[86] Deignan J.L., Chung W.K., Kearney H.M., Monaghan K.G., Rehder C.W., Chao E.C. (2019). Points to consider in the reevaluation and reanalysis of genomic test results: A statement of the American College of Medical Genetics and Genomics. Genet. Med..

[87] Wright C.F., Campbell P., Eberhardt R.Y., Aitken S., Perrett D., Brent S., Danecek P., Gardner E.J., Chundru V.K., Lindsay S.J. (2023). Genomic Diagnosis of Rare Pediatric Disease in the United Kingdom and Ireland. N. Engl. J. Med..

[88] Manrai A.K., Funke B.H., Rehm H.L., Olesen M.S., Maron B.A., Szolovits P., Margulies D.M., Loscalzo J., Kohane I.S. (2016). Genetic Misdiagnoses and the Potential for Health Disparities. N. Engl. J. Med..

[89] Botkin J.R., Belmont J.W., Berg J.S., Berkman B.E., Bombard Y., Holm I.A., Levy H.P., Ormond K.E., Saal H.M., Spinner N.B. (2015). Points to Consider: Ethical, Legal, and Psychosocial Implications of Genetic Testing in Children and Adolescents. Am. J. Hum. Genet..

[90] Lee K., Abul-Husn N.S., Amendola L.M., Brothers K.B., Chung W.K., Gollob M.H., Gordon A.S., Harrison S.M., Hershberger R.E., Li M. (2025). ACMG SF v3.3 list for reporting of secondary findings in clinical exome and genome sequencing: A policy statement of the American College of Medical Genetics and Genomics. Genet. Med..

[91] Wojcik M.H., Lemire G., Berger E., Zaki M.S., Wissmann M., Win W., White S.M., Weisburd B., Wieczorek D., Waddell L.B. (2024). Genome Sequencing for Diagnosing Rare Diseases. N. Engl. J. Med..

[92] Smedley D., Smith K.R., Martin A., Thomas E.A., McDonagh E.M., Cipriani V., Ellingford J.M., Arno G., Tucci A., Vandrovcova J. (2021). 100,000 Genomes Pilot on Rare-Disease Diagnosis in Health Care—Preliminary Report. N. Engl. J. Med..

[93] AlAbdi L., Shamseldin H.E., Khouj E., Helaby R., Aljamal B., Alqahtani M., Almulhim A., Hamid H., Hashem M.O., Abdulwahab F. (2023). Beyond the exome: Utility of long-read whole genome sequencing in exome-negative autosomal recessive diseases. Genome Med..

[94] Park J., Shrestha R., Qiu C., Kondo A., Huang S., Werth M., Li M., Barasch J., Suszták K. (2018). Single-cell transcriptomics of the mouse kidney reveals potential cellular targets of kidney disease. Science.

[95] Lunke S., Bouffler S.E., Patel C.V., Sandaradura S.A., Wilson M., Pinner J., Hunter M.F., Barnett C.P., Wallis M., Kamien B. (2023). Integrated multi-omics for rapid rare disease diagnosis on a national scale. Nat. Med..

[96] Cummings B.B., Marshall J.L., Tukiainen T., Lek M., Donkervoort S., Foley A.R., Bolduc V., Waddell L.B., Sandaradura S.A., O’Grady G.L. (2017). Improving genetic diagnosis in Mendelian disease with transcriptome sequencing. Sci. Transl. Med..

[97] Hirayama R., Toyohara K., Watanabe K., Otsuki T., Araoka T., Mae S.-I., Horinouchi T., Yamamura T., Okita K., Hotta A. (2023). iPSC-derived type IV collagen α5-expressing kidney organoids model Alport syndrome. Commun. Biol..

[98] Bergquist T., Stenton S.L., Nadeau E.A.W., Byrne A.B., Greenblatt M.S., Harrison S.M., Tavtigian S.V., O’Donnell-Luria A., Biesecker L.G., Radivojac P. (2025). Calibration of additional computational tools expands ClinGen recommendation options for variant classification with PP3/BP4 criteria. Genet. Med..

[99] Bassanese G., Wlodkowski T., Servais A., Heidet L., Roccatello D., Emma F., Levtchenko E., Ariceta G., Bacchetta J., Capasso G. (2021). The European Rare Kidney Disease Registry (ERKReg): Objectives, design and initial results. Orphanet J. Rare Dis..

